# Multiple Human Population Movements and Cultural Dispersal Events Shaped the Landscape of Chinese Paternal Heritage

**DOI:** 10.1093/molbev/msae122

**Published:** 2024-06-17

**Authors:** Mengge Wang, Yuguo Huang, Kaijun Liu, Zhiyong Wang, Menghan Zhang, Haibing Yuan, Shuhan Duan, Lanhai Wei, Hongbing Yao, Qiuxia Sun, Jie Zhong, Renkuan Tang, Jing Chen, Yuntao Sun, Xiangping Li, Haoran Su, Qingxin Yang, Liping Hu, Libing Yun, Junbao Yang, Shengjie Nie, Yan Cai, Jiangwei Yan, Kun Zhou, Chuanchao Wang, 10K_CPGDP Consortium, Guanglin He, Chao Liu, Mengge Wang, Renkuan Tang, Libing Yun, Junbao Yang, Chuan-Chao Wang, Jiangwei Yan, Bofeng Zhu, Liping Hu, Shengjie Nie, Hongbing Yao, Bofeng Zhu, Chao Liu, Guanglin He

**Affiliations:** Institute of Rare Diseases, West China Hospital of Sichuan University, Sichuan University, Chengdu 610000, China; Center for Archaeological Science, Sichuan University, Chengdu 610000, China; Faculty of Forensic Medicine, Zhongshan School of Medicine, Sun Yat-sen University, Guangzhou 510275, China; Institute of Rare Diseases, West China Hospital of Sichuan University, Sichuan University, Chengdu 610000, China; School of International Tourism and Culture, Guizhou Normal University, Guiyang 550025, China; MoFang Human Genome Research Institute, Tianfu Software Park, Chengdu, Sichuan 610042, China; Institute of Rare Diseases, West China Hospital of Sichuan University, Sichuan University, Chengdu 610000, China; School of Forensic Medicine, Kunming Medical University, Kunming 650500, China; Institute of Modern Languages and Linguistics, Fudan University, Shanghai 200433, China; Research Institute of Intelligent Complex Systems, Fudan University, Shanghai 200433, China; Center for Archaeological Science, Sichuan University, Chengdu 610000, China; Institute of Rare Diseases, West China Hospital of Sichuan University, Sichuan University, Chengdu 610000, China; School of Basic Medical Sciences, North Sichuan Medical College, Nanchong 637100, China; School of Ethnology and Anthropology, Institute of Humanities and Human Sciences, Inner Mongolia Normal University, Hohhot 010022, China; Belt and Road Research Center for Forensic Molecular Anthropology Gansu University of Political Science and Law, Lanzhou 730000, China; Institute of Rare Diseases, West China Hospital of Sichuan University, Sichuan University, Chengdu 610000, China; Department of Forensic Medicine, College of Basic Medicine, Chongqing Medical University, Chongqing 400331, China; Institute of Rare Diseases, West China Hospital of Sichuan University, Sichuan University, Chengdu 610000, China; Department of Forensic Medicine, College of Basic Medicine, Chongqing Medical University, Chongqing 400331, China; Institute of Rare Diseases, West China Hospital of Sichuan University, Sichuan University, Chengdu 610000, China; School of Forensic Medicine, Shanxi Medical University, Jinzhong 030001, China; Institute of Rare Diseases, West China Hospital of Sichuan University, Sichuan University, Chengdu 610000, China; Institute of Forensic Medicine, West China School of Basic Medical Sciences & Forensic Medicine, Sichuan University, Chengdu 610041, China; Institute of Rare Diseases, West China Hospital of Sichuan University, Sichuan University, Chengdu 610000, China; School of Forensic Medicine, Kunming Medical University, Kunming 650500, China; Institute of Rare Diseases, West China Hospital of Sichuan University, Sichuan University, Chengdu 610000, China; School of Laboratory Medicine and Center for Genetics and Prenatal Diagnosis, Affiliated Hospital of North Sichuan Medical College, Nanchong, Sichuan 637007, China; Institute of Rare Diseases, West China Hospital of Sichuan University, Sichuan University, Chengdu 610000, China; School of Forensic Medicine, Kunming Medical University, Kunming 650500, China; School of Forensic Medicine, Kunming Medical University, Kunming 650500, China; Institute of Forensic Medicine, West China School of Basic Medical Sciences & Forensic Medicine, Sichuan University, Chengdu 610041, China; Institute of Basic Medicine and Forensic Medicine, North Sichuan Medical College and Center for Genetics and Prenatal Diagnosis, Affiliated Hospital of North Sichuan Medical College, Nanchong, Sichuan 637007, China; School of Forensic Medicine, Kunming Medical University, Kunming 650500, China; School of Laboratory Medicine and Center for Genetics and Prenatal Diagnosis, Affiliated Hospital of North Sichuan Medical College, Nanchong, Sichuan 637007, China; School of Forensic Medicine, Shanxi Medical University, Jinzhong 030001, China; MoFang Human Genome Research Institute, Tianfu Software Park, Chengdu, Sichuan 610042, China; State Key Laboratory of Cellular Stress Biology, School of Life Sciences, Xiamen University, Xiamen 361005, China; Guangzhou Key Laboratory of Forensic Multi-Omics for Precision Identification, School of Forensic Medicine, Southern Medical University, Guangzhou 510515, China; Microbiome Medicine Center, Department of Laboratory Medicine, Zhujiang Hospital, Southern Medical University, Guangzhou, Guangdong 510515, China; Guangzhou Key Laboratory of Forensic Multi-Omics for Precision Identification, School of Forensic Medicine, Southern Medical University, Guangzhou 510515, China; Anti-Drug Technology Center of Guangdong Province, Guangzhou 510230, China; Institute of Rare Diseases, West China Hospital of Sichuan University, Sichuan University, Chengdu 610000, China; Center for Archaeological Science, Sichuan University, Chengdu 610000, China

**Keywords:** YanHuang cohort, Y-chromosome phylogeny, evolutionary history, founding lineage

## Abstract

Large-scale genomic projects and ancient DNA innovations have ushered in a new paradigm for exploring human evolutionary history. However, the genetic legacy of spatiotemporally diverse ancient Eurasians within Chinese paternal lineages remains unresolved. Here, we report an integrated Y-chromosome genomic database encompassing 15,563 individuals from both modern and ancient Eurasians, including 919 newly reported individuals, to investigate the Chinese paternal genomic diversity. The high-resolution, time-stamped phylogeny reveals multiple diversification events and extensive expansions in the early and middle Neolithic. We identify four major ancient population movements, each associated with technological innovations that have shaped the Chinese paternal landscape. First, the expansion of early East Asians and millet farmers from the Yellow River Basin predominantly carrying O2/D subclades significantly influenced the formation of the Sino-Tibetan people and facilitated the permanent settlement of the Tibetan Plateau. Second, the dispersal of rice farmers from the Yangtze River Valley carrying O1 and certain O2 sublineages reshapes the genetic makeup of southern Han Chinese, as well as the Tai-Kadai, Austronesian, Hmong-Mien, and Austroasiatic people. Third, the Neolithic Siberian Q/C paternal lineages originated and proliferated among hunter-gatherers on the Mongolian Plateau and the Amur River Basin, leaving a significant imprint on the gene pools of northern China. Fourth, the J/G/R paternal lineages derived from western Eurasia, which were initially spread by Yamnaya-related steppe pastoralists, maintain their presence primarily in northwestern China. Overall, our research provides comprehensive genetic evidence elucidating the significant impact of interactions with culturally distinct ancient Eurasians on the patterns of paternal diversity in modern Chinese populations.

## Introduction

Population genomics and human pangenome projects aim to comprehensively document the genetic landscapes of globally diverse populations, elucidate their demographic histories, and uncover the genetic underpinnings of complex traits and diseases (Bergstrom et al. 2020; Byrska-Bishop et al. 2022). East Asia serves as one of the earliest cradles of civilization and the crossroads of the peopling of Oceania, Siberia, and America, whose genetic landscape is poorly characterized in the era of population genomics. China harbors extensive genetic, physical, cultural, and ethnolinguistic diversities, positioning it uniquely for studying the intricate demographic histories of diverse populations, including human divergence, migration, and admixture, and the interplay between genetics and culture (Wang et al. 2021b; Kumar et al. 2022). Numerous studies have sought to bridge the knowledge gap regarding the genetic diversity of Chinese populations by examining their evolutionary histories and the genetics of complex traits and diseases. Recent research utilized genome-wide SNP microarrays to analyze the genomic diversity and population history of various Sino-Tibetan, Mongolic, Tungusic, Turkic, Tai-Kadai, and Hmong-Mien groups (Feng et al. 2017; He et al. 2022; Wang et al. 2022; He et al. 2023b; Sun et al. 2023; Li et al. 2024). Additionally, the rise of whole-genome sequencing studies has expanded, featuring projects, such as the Westlake BioBank for Chinese, the NyuWa genome resource, the China Metabolic Analytics Project, and the 10K Chinese People Genomic Diversity Project (10K_CPGDP; Cao et al. 2020; Zhang et al. 2021a; Cong et al. 2022; Cheng et al. 2023; He et al. 2023c). These efforts enhance our understanding of the genetic diversity, demographic history, and genetic architecture of complex traits and diseases in ethnolinguistically distinct Chinese populations from an autosomal perspective, suggesting a further exploration of their fine-scale genetic structure from both uniparental and population-scale project perspectives.

The nonrecombining portion of the Y-chromosome has become pivotal in studying human evolutionary history across various time scales (Poznik et al. 2016). Recent advancements in sequencing technologies and computational methods for genome assembly, read mapping, variant calling, and benchmarking have significantly improved the generation of complete Y-chromosome sequences, enriching our understanding of Y-chromosome variations (Olson et al. 2023). These developments have facilitated the construction of a robust phylogenetic tree, with branch lengths indicating mutation counts (Poznik et al. 2016; Zhabagin et al. 2022). Over the past two decades, studies on targeted Y-SNPs have traced ancestral lines through paternal lineages, providing crucial phylogenetic data for research on human origins, migrations, and admixture (Su et al. 1999; Zerjal et al. 2003). Resequencing the entire Y-chromosome region using advanced next-generation sequencing and computational techniques has transformed research paradigms. For instance, Wei et al. identified 6,662 high-confidence variants across 36 diverse Y-chromosome sequences, refining existing Y-chromosome phylogenies (Wei et al. 2013). Similarly, Poznik et al. (2016) analyzed 1,244 complete Y-chromosome genomes from the 1000 Genomes Project (1KGP), uncovering over 65,000 variants and identifying recent expansions within specific paternal lineages. Studies on single populations or specific lineages have also been conducted. The O1a-M119 lineage, which is shared among the Sinitic, Tai-Kadai, and Austronesian groups, and key paternal lineages like C2a-F5484 and Q1a1a-M120 have been examined to trace their origins, diffusion, and contributions to the gene pools of Chinese ethnolinguistically diverse groups (Sun et al. 2019; Wu et al. 2020; Sun et al. 2021). However, the availability of large-scale Y-chromosome genomic databases for China remains limited, underscoring the need for more comprehensive databases to explore the paternal genetic landscape and its historical influences on diverse populations.

Recent increases in genomic resources from Chinese populations have highlighted the gap in our understanding of the paternal genetic diversity among ethnic minorities, which lags significantly behind that of Han Chinese and other global populations (Karmin et al. 2022). To address this issue, we launched the 10K_CPGDP by employing anthropologically informed sampling strategies (He et al. 2023c). Additionally, we introduce the YanHuang cohort (YHC) genomic resource that includes new Y-chromosome sequences from ethnolinguistically diverse ethnic minorities and integrates data from the 10K_CPGDP. The YHC aims to provide a high-quality population-specific Y-chromosome database, delineate the fine-scale paternal demographic history of underrepresented groups, construct a high-resolution, time-stamped phylogenetic tree, and develop novel East Asian-specific next-generation sequencing panels covering SNPs, STRs, InDels, and other variants for medical and forensic use. We also developed the “YHSeqY3000”, the highest-resolution Y-specific targeted resequencing panel designed from whole-genome and genome-wide SNP data of Y-chromosomes within the YHC. We genotyped 2,999 panel-related Y-SNPs in 919 males from 57 diverse ethnic minorities who were also genotyped by whole Y-chromosome sequencing. Our efforts culminated in a comprehensive Y-chromosome database encompassing 15,563 individuals from modern and ancient Eurasian backgrounds, allowing us to construct the first fully resolved phylogeny incorporating ancient DNA sequences. This phylogeny helps estimate the coalescence dates of dominant lineages, trace the origins of Chinese paternal lineages, and elucidate the impacts of historical migrations, admixture, and shifts in subsistence strategies on the genetic architecture of these diverse groups.

## Results and Discussion

### Genetic Diversity of YHC Paternal Lineages Inferred from Y-Chromosome Sequences and the YHSeqY3000 Panel

**Fig. 1.**
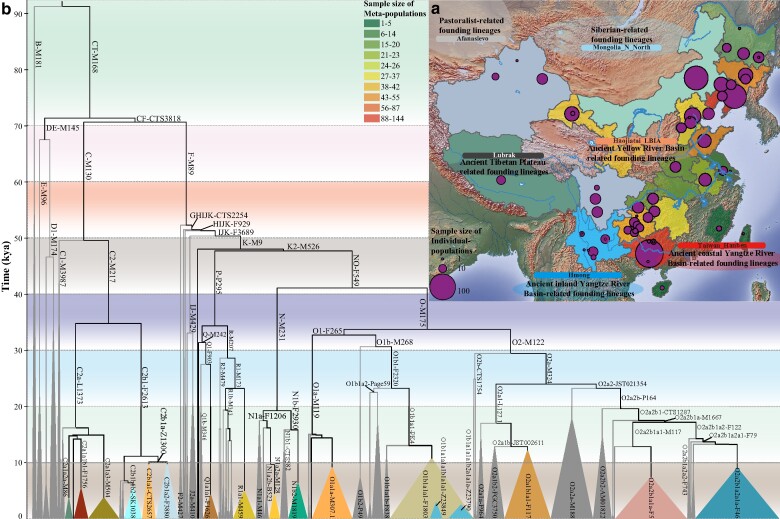
Geographic location and phylogenetic characteristics of 919 newly sequenced Chinese minority individuals. a) A map of East Asia displays essential data for 919 individuals from 57 Chinese ethnic minority groups. Circle sizes on the map indicate the sample sizes of individual populations, while colored provinces represent sampling locations, with colors denoting the total sample size from those regions. Additionally, ancient subsistence strategies, such as pastoralism, hunter-gathering, and agriculture, from western Eurasia, the Mongolian Plateau, and the origin centers of Chinese agriculture in the Yellow and Yangtze River basins are depicted. b) The Y-chromosome phylogeny includes 914 individuals who passed quality control, illustrating the most recent common ancestors (TMRCA) of various prevalent paternal lineages. B-lineage-related representative haplotypes from the Simons Genome Diversity Project serve as an outgroup. Branch lengths correlate with the estimated TMRCA. Major lineages are indicated by colored triangles, with the base width of each triangle proportional to the sample size. A detailed, time-stamped phylogenetic tree is presented in supplementary fig. S1, Supplementary Material online, with scales of divergence times differentiated by varying background colors.

We performed whole Y-chromosome sequencing on 919 participants from 57 populations of 39 ethnic minorities (Fig. 1a; supplementary table S1, Supplementary Material online), integrated the genetic data of nearly 15,000 modern and ancient Eurasian people (supplementary tables S2 and S3, Supplementary Material online), and developed a high-resolution YHSeqY3000 panel, including Y-SNPs not present in existing phylogenetic databases (ISOGG, Yfull). The predominant paternal lineages identified, namely, C-M130, N-M231, O-M175, and R-M207, demonstrated haplogroup frequencies greater than 5% (supplementary fig. S1 and table S4, Supplementary Material online). Additional sublineages, such as D1-M174 and E1-P147, were also noted among these minorities (supplementary fig. S1 and table S4, Supplementary Material online). For the haplogroup classification, three methods were used, namely, in-house scripts, Y-LineageTracker, and HaploGrouper, to simultaneously infer haplogroups from the YHSeqY3000 panel data. The discrepancies in the classification results highlighted the need for improved accuracy in the haplogroup determination, especially the 40 significant discrepancies involving major subclades like C-M130 and J-M304 based on the Y-LineageTracker classification (supplementary table S4, Supplementary Material online). In contrast, the haplogroup differences obtained based on HaploGrouper were minimal (supplementary table S4, Supplementary Material online). The analysis revealed 564 distinct paternal lineages, with 384 subhaplogroups observed only once (supplementary fig. S2 and table S4, Supplementary Material online). This underpins the necessity for a continuous refinement of Y-chromosome phylogenetic trees to accommodate newly identified Y-SNPs and update the haplogroup classification tool (Chen et al. 2021; Jagadeesan et al. 2021). The upcoming version of the YHC phylogenetic topology aims to address these gaps. Overall, the resolution and coverage of the YHSeqY3000 panel confirmed by the minimal differences in the haplogroup classification compared to ∼10 Mb Y-chromosome sequences establish it as the most refined system to date for high-resolution paternal lineage analysis in Chinese populations (supplementary table S4, Supplementary Material online). This system exceeds the capabilities of previous methods, ensuring a more precise haplogroup classification at a finer scale (Wang et al. 2019; He et al. 2023a).

### Genetic Connections and Population Stratification among Modern and Ancient Eurasians

We explored the population differentiation among spatiotemporally diverse Eurasian populations based on the clustering patterns identified via the principal component analysis (PCA), multidimensional scaling analysis (MDS), and other population genetic analyses. The PCA distinctly separated ancient western Eurasians from East Asians, with each group exhibiting unique patterns of dominant paternal lineages and clustering branches on the phylogenetic tree (supplementary fig. S3a to j, Supplementary Material online). Modern population clustering aligns with their geographic and linguistic attributes, showing a clear separation among most Austronesian and Tibeto-Burman groups, while other populations demonstrate a considerable overlap in their clustering positions (supplementary fig. S4, Supplementary Material online). Iron Age (IA) Hanben individuals show close genetic ties with Austronesian groups, and northern Chinese individuals are closely aligned with Sino-Tibetan groups. Notably, there is a marked stratification between northern and southern East Asians, with further substructures among linguistically similar, but geographically distinct groups (supplementary figs. S5 to S7, Supplementary Material online). For instance, IA Hanben populations align closely with modern Han populations from Guangxi and Taiwan, whereas Yellow River Basin farmers form distinct clusters from other Han groups (supplementary fig. S7a, Supplementary Material online). Diverse Tibeto-Burman groups exhibited genetic distinctions between their northern and southern divisions (supplementary fig. S7b, Supplementary Material online). A significant differentiation was also evident among the Transeurasian-speaking groups, with the Koreanic and Japonic groups forming separate clades, the Mongolic and some Tungusic groups clustering together, and the Turkic groups sharing close affinity with certain Tungusic populations (supplementary fig. S5, Supplementary Material online). In South China and Southeast Asia (SEA), fine-scale clustering among the Austroasiatic, Austronesian, Hmong-Mien, and Tai-Kadai groups suggests an extensive gene flow, as evidenced by their overlapping genetic patterns (supplementary fig. S6, Supplementary Material online). Phylogenetic relationships and haplogroup frequency spectra highlighted genetic disparities between northern and southern Han groups and between northern and southern Tibeto-Burman speakers, while the gene flow was apparent between geographically proximate groups, such as between Austronesian and southern Han populations and between Transeurasian and northern Han populations (supplementary fig. S8, Supplementary Material online). This comprehensive analysis elucidates the complex genetic landscape and interactions among Eurasian populations.

We grouped populations by linguistic and ethnic traits to investigate genetic affinities within language- or ethnicity-based metapopulations (supplementary fig. S7c to h, Supplementary Material online). Geographically close populations, including the Austronesian-speaking Saisiyat, Thao, Taroko, Atayal, and Tsou from Taiwan Province, clustered distinctly, separating early from other reference groups (supplementary fig. S7c, Supplementary Material online). Distinct branches primarily comprised Tai-Kadai, nearby Austronesian groups like Ede and Giarai, and southern Tibeto-Burman speakers, such as Sila and Lolo. The genetic closeness between the Austronesian-related and Tai-Kadai-dominant clusters supports the hypothesis of a shared origin for Austronesian and Tai-Kadai speakers, as demonstrated by phylogenetic analyses based on neighbor-joining methods and clustering inferred from the haplogroup frequency spectra, PCA, and MDS (supplementary fig. S7d to f, Supplementary Material online). These analyses also revealed fine-scale genetic differences between Han Chinese and Tibeto-Burman populations and among linguistically diverse groups, underscoring frequent massive population movements and gene flow events in historical contexts. To determine whether paternal lineages corroborate current language family classifications and further explore genetic relationships within linguistically defined metapopulations, we merged all groups based on linguistic affinities for a comprehensive population genetic analysis (supplementary fig. S7g and h, Supplementary Material online). Notably, a close genetic clustering between the Tai-Kadai and Austroasiatic groups and between the Mongolic/Tungusic groups and the Amur River Basin ancient populations was observed. The neighbor-joining tree also indicated close genetic relationships between the Turkic and ancient Xinjiang populations, between the Koreanic and Japonic populations, and between the Austronesian and ancient Hanben populations (supplementary fig. S7h, Supplementary Material online). This study provides robust paternal genetic evidence supporting complex admixture and interactions among modern Chinese populations and ancient Eurasians. However, caution is advised regarding potential biases from low-coverage sampling and the simplistic grouping of linguistically similar, yet geographically disparate populations.

### Complex Population Migration and Admixture Events Inferred from the Y-Chromosome Diversity Landscape

**Fig. 2.**
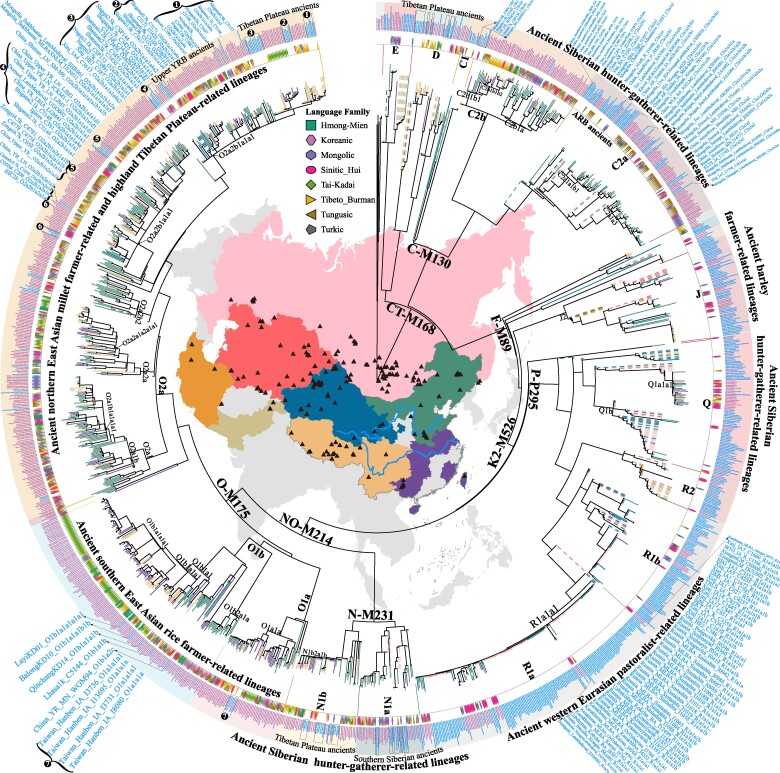
Maximum likelihood phylogenetic tree among modern and ancient Eurasian populations. This tree includes newly genotyped individuals from Chinese ethnic minorities and ancient Eurasian reference populations. The colored regions on the map signify the sampling locations of both ancient and modern Eurasian populations, with black triangles marking the sites where ancient Eurasian samples were collected. The branch lengths are proportional to the mutation counts, and the branch colors indicate the sampling locations, with solid lines representing modern individuals and dashed lines depicting ancient individuals. Inside the circle, different shapes and colors denote the language families of modern individuals: rosy for Sinitic; dark goldenrod for Tibeto-Burman; bluish violet for Mongolic; brown for Tungusic; gray for Turkic; lavender for Koreanic; green for Tai-Kadai; and medium sea green for Hmong-Mien. Due to the small sample sizes (fewer than five), Austronesian, Austroasiatic, and Indo-European-speaking populations are not labeled. The outer circle displays sample information and corresponding haplogroup results, with violet representing modern individuals and light blue for ancient ones. Enlargements of dominant lineages and their representative ancient genomes are highlighted at the four corners of the map. Detailed views of these branches are provided in supplementary figs. S9 to S12, Supplementary Material online.

**Fig. 3.**
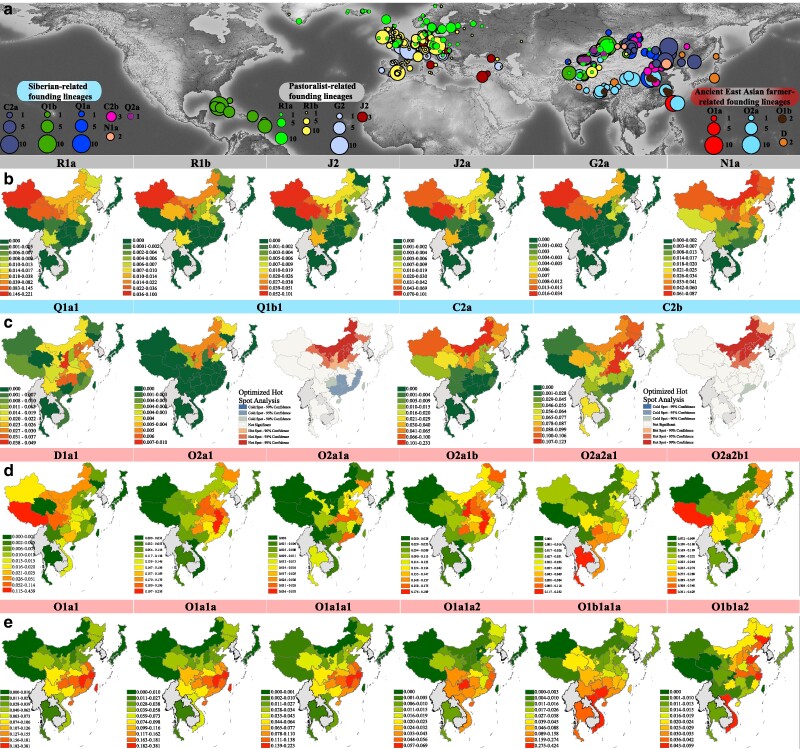
Frequency spectrum of dominant Chinese paternal lineages in ancient Eurasians and modern East and Southeast Asians. a) The geographic distribution of 1,284 ancient individuals carrying 12 Y-chromosome lineages is depicted. Various haplogroups are represented by circles of different colors, with the size of each circle proportional to the frequency of the corresponding haplogroups. b and c) Frequencies of paternal lineages related to Western-origin and Siberian hunter-gatherers among eastern Eurasian populations are shown. Optimized hot spot analysis was employed to suggest the geographic origins of these focused lineages. d and e) Frequencies of sublineages associated with early East Asian-related D, ANEA millet farmer-related O2, and ancient Southern East Asian rice farmer-related O1 are displayed. Areas with high frequencies or significant phylogeographic relevance of the studied lineages are highlighted in hot red. Detailed frequency distributions of additional sublineages are provided in supplementary figs. S13 and S16 to S18, Supplementary Material online.

**Fig. 4.**
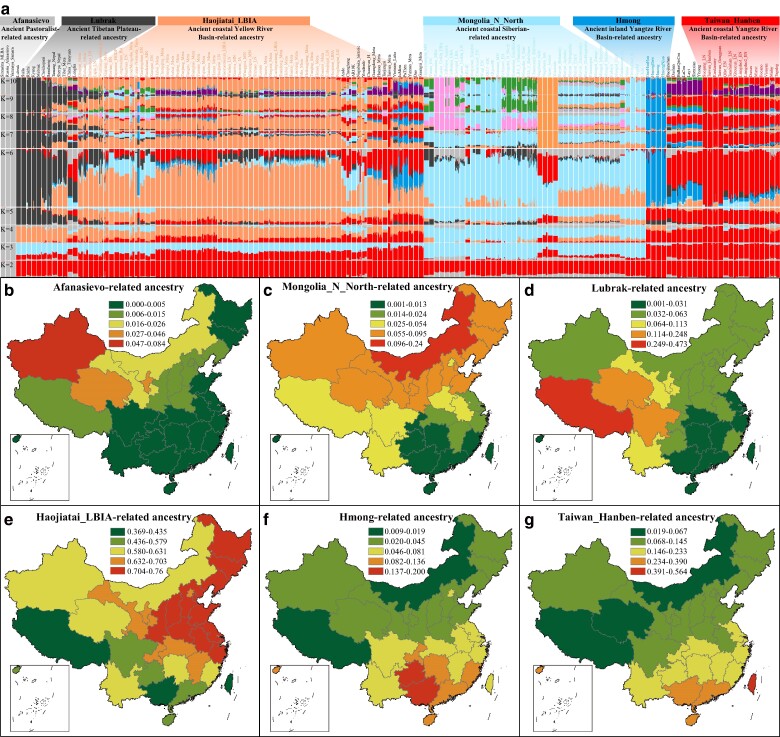
Admixture results and geographic distribution of ancestral sources. a) Model-based ADMIXTURE analysis was performed for modern and ancient East Asian populations using predefined ancestral sources ranging from 2 to 15. The optimal fit was achieved with a six-way admixture model, which exhibited the lowest cross-validation error. b to g) The distribution of admixture proportions across various Chinese populations is depicted, with red indicating the highest proportion of a specific ancestral component.

**Fig. 5.**
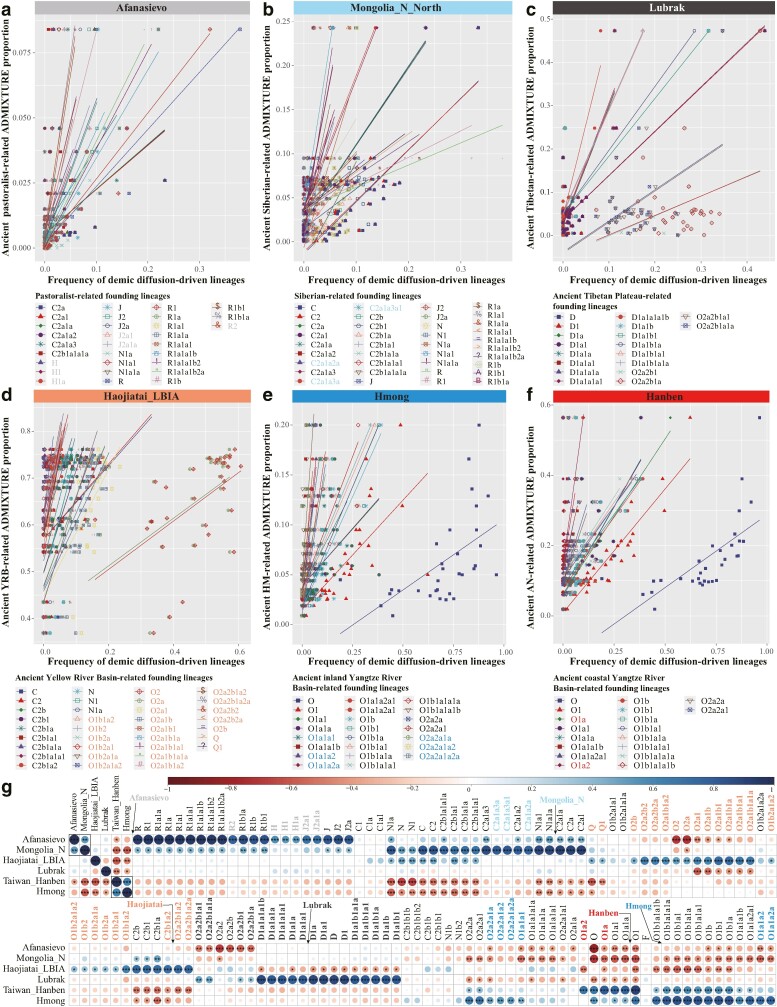
Correlation between the autosomal ancestral proportions and the frequencies of the Y-chromosome lineages. a to f) Scatter plots display statistically significant positive correlations between autosomal-based ancestral proportions and population-specific founding paternal lineages. g) Correlations between autosomal-based admixture estimates of ancestral proportions and frequencies of paternal lineages are shown. Correlations involving proportions of different ancestral sources were excluded, and the initial clustering positions of these correlations are marked with arrows. This visual analysis elucidates the complex interplay between autosomal and Y-chromosomal data in tracing genetic heritage and lineage dynamics.

The observed paternal genetic structure indicated that multiple complex ancient migration and admixture events significantly shaped the gene pool of Chinese populations. A time-stamped phylogenetic tree revealed multiple lineage diversifications after the last glacial maximum (20 kya), with these lineages dispersing at varying times (Fig. 1b; supplementary fig. S1, Supplementary Material online). Analysis using a maximum likelihood (ML) tree incorporating ancient DNA sequences revealed diverse founding populations contributing to the Chinese paternal gene pool that likely originated from ancient migrations of descendants from indigenous rice or millet farmers, Siberian hunter-gatherers, or western Eurasian steppe pastoralists (Fig. 2; supplementary figs. S9 to S12, Supplementary Material online). The extent to which ancestral sources affected the paternal genetic makeup of Chinese ethnic minorities was systematically investigated, along with the geographical spread of identified lineages and their associations with expansions related to ancient farmers, hunter-gatherers, and pastoralists. Additionally, to determine the origins and distribution patterns of dominant paternal lineages in China, the participants were grouped into geographically defined metapopulations, and general geographical distribution patterns were estimated (Figs. 3 to 5; supplementary figs. S17 and S18, Supplementary Material online). Finally, we systematically assessed how ancient technological innovations and human migration events have influenced the paternal genetic landscape of Chinese populations, revealing a complex interplay of genetic inputs from various ancient populations.

#### Gene Flow from Ancient Pastoralists and Barley Farmers in West Eurasia and Central/South Asia to East Asia

Prehistoric and historical cultural exchanges along the southern Bactrian Marianna Archaeological Complex oasis farming route, Inner Asian Mountain Corridor, and northern Yamnaya/Afanasievo steppe pastoralist migration routes have significantly shaped the autosomal gene pool of ancient populations in the Altai Mountains and surrounding areas of northwestern and northern East Asia (Zhang et al. 2021b). Haplogroups J/G/R and their major sublineages, which are prevalent among ancient western Eurasians, exhibit the highest frequencies in Northwest China (Figs. 2 and 3a and b; supplementary fig. S9a, Supplementary Material online). Specifically, most J haplogroup carriers in China belong to the J2-M172 sublineage, particularly J2a-M410. The origins of J2a in ancient populations can likely be traced back to the northern Fertile Crescent, and its current distribution primarily reflects expansions and admixture events related to ancient barley farmers (Figs. 2 and 3a). Similarly, individuals carrying G-M201 in Northwest China were predominantly classified under sublineage G2a (Fig. 3b). An optimized hot spot analysis revealed diffusion centers for J2a and G2a in the Xinjiang and Gansu–Qinghai regions, suggesting a correlation with these areas (supplementary fig. S17a, Supplementary Material online). Generally, the introduction of J/G-derived lineages into China is attributed to the eastward migration of barley farmer-related ancestral populations likely facilitated by gene flow events along the ancient Silk Road (Zhabagin et al. 2022; He et al. 2023c).

R-M207 is predominantly found among ancient western Eurasians and modern populations in North China, particularly in Northwest China (Figs. 2 and 3a and b). The basal haplogroup R was identified in a ∼24,000-year-old individual from the Mal’ta site near Lake Baikal in Siberia (Raghavan et al. 2014). In China, approximately 90% of R carriers are categorized as R1-M173, which bifurcates into R1a-L146 and R1b-M343 approximately 23 kya. The frequency of R1a-L146 notably exceeded that of R1b-M343 (Figs. 1b and 3b; supplementary fig. S1, Supplementary Material online). Furthermore, all individuals within R1a were classified into R1a1a sublineages, with R1a1a1b diverging approximately 5 kya and being the most prevalent (Fig. 1b; supplementary fig. S1 and table S5, Supplementary Material online). The spatiotemporal distribution of R1 subclades is closely linked to the movements of ancient steppe pastoralists, underscoring a significant genetic flow into China (Figs. 2 and 3a). Conversely, R2-M479 appears in East Asia at low frequencies (supplementary fig. S17a, Supplementary Material online) and is primarily concentrated in Central/South Asia, having recently extended from South Asia to North China via the northern route. Analysis combining ancient and modern population phylogenies revealed that samples from Mongolia with substantial West Eurasian ancestry, such as Mongolia_EIA_Sagly_4 and Mongolia_LBA_MongunTaiga_3, fall within the R1a1a sublineage. Nearly half of the ancient Xinjiang individuals are categorized within sublineages R1a or R1b, reflecting the historical impact of the Yamnaya/Afanasievo-related pastoralists on the genetic makeup of the northwestern Chinese populations (Figs. 2 and 3a). Additionally, the sporadic presence of other rare haplogroups like H-L901 and I-M170 in China suggests a broad and recent gene flow from Central/South Asian and West Eurasian ancestors into the region.

To confirm that migrations related to pastoralist populations have reshaped the distribution of western Eurasian-related lineages in Chinese populations, we estimated the correlation between haplogroup frequencies and both geographical (longitude and latitude) and genetic features (PC1-2, haplogroup frequency, Fst matrix, and autosomal-based admixture proportions). The frequencies of R-related sublineages correlate with latitude and exhibit high frequency in modern northwestern Chinese populations (supplementary fig. S14a and b, Supplementary Material online). Furthermore, the distribution patterns of R and its sublineages were significantly correlated (supplementary fig. S14c, Supplementary Material online). To elucidate the direct genetic contributions from ancient sources to modern Chinese populations, we constructed a six-source admixture model, revealing a gradual decrease in ancestral proportions from their archeologically confirmed origins or earliest emergence areas in China (Fig. 4b). If ancient migration events directly influence the lineage frequency patterns in Chinese populations, a solid positive correlation would be expected between the proportion of autosomal-based admixture from presumed ancestral sources and the frequency of founding lineages. Intriguingly, a significant correlation was observed between the Afanasievo-related ancestral proportions and the haplogroup frequencies of multiple H, J, and R sublineages (Fig. 5a and g). These findings, derived from the haplogroup frequency spectra of modern and ancient Eurasians, phylogeographic origin inferences, and multiple factor correlations, suggest that migrations of western Eurasian barley and pastoralist-related populations likely facilitated the development of these Chinese founding lineages.

#### Siberian Hunter-Gatherer-Dominant Paternal Lineages Are Widely Distributed in China

Ancient DNA studies have identified an ancestral component, termed Ancient Northeast Asian (ANA) ancestry, related to Neolithic hunter-gatherers from the Russian Far East, Mongolian Plateau, and Baikal region (Jeong et al. 2020; Fig. 4a and c). This ANA-related ancestry has contributed variably to distinct ancient populations in these regions, which are characterized by high proportions of C/N/Q/R sublineages (Figs. 2 and 3a). The frequencies of the C2/N1/R1 sublineages were significantly positively correlated with the ANA-related ancestry (*P* < 0.05, Fig. 5b and g). The haplogroup Q-M242 appears in China at very low frequencies (<3%, supplementary table S5, Supplementary Material online) and displays varied distribution patterns between North and South China (Fig. 3c; supplementary table S5, Supplementary Material online). This lineage, which might have originated in Central Asia and southern Siberia approximately 31 kya (Fig. 1b), includes the Q1a1a-M120 subclade. This subclade, unique to East Asians, is relatively prevalent among Han Chinese individuals (∼81% of all Q lineages, supplementary table S5, Supplementary Material online) and likely underwent a local expansion in Northwest China between 5 and 3 kya (Sun et al. 2019). Furthermore, the Q1a1a1-F1626 subclade, a derivative of Q1a1a-M120, diversified approximately 4.3 kya (Fig. 1b). The ML phylogenetic topology indicated that ancient Mongolian individuals with minimal West Eurasian-related ancestry (<20%) belonged to Q1a1a or its sublineages (Figs. 2 and 3a). Venn diagrams illustrating shared ancestry-correlated lineages also show that the Q and R lineages are common among the Yamnaya and ANA-associated lineages (supplementary fig. S15, Supplementary Material online). Moreover, ancient individuals from the middle Neolithic (MN) Yangshao culture and approximately 3,000-year-old Hengbei residents from Shanxi, who carried the Q1a1a-M120 lineage, indicate that this haplogroup influenced the Han Chinese gene pool at least 6 kya. Q1b-M346, although rare in China, is concentrated at the intersection of Siberia and North China (supplementary fig. S17b and table S5, Supplementary Material online), with some Bronze Age (BA) and IA individuals from the Mongolian Plateau and Xinjiang regions genotyped for Q1b or its subclades (Figs. 2 and 3a).

Haplogroup N-M231, particularly its subclade N1-CTS3750, is prevalent among Chinese populations, diverging into N1a-F1206 and N1b-F2930 around 19 kya (Fig. 1b; supplementary fig. S1, Supplementary Material online). Y-chromosome analyses of ancient individuals from the West Liao River Basin, dating from 6,500 to 2,700 BP, indicated that N-M231 was the dominant paternal lineage in Northeast China during the Neolithic, with its frequency gradually declining over time (Cui et al. 2013). The frequencies of the N1a-F1206 and N1b-F2930 subclades were high in North China and low-altitude Southwest China, respectively (supplementary fig. S17b, Supplementary Material online). These findings suggest a north‒south differentiation of N1 subclades in North China, with N1a-F1206 migrating northward beyond East Asia and N1b-F2930 moving southward to become a major paternal lineage among Tibeto-Burman groups, notably the Yi people (supplementary table S5, Supplementary Material online). N1a1-M46/Tat, a dominant subclade, likely originated in Northeast Asia. An individual from the Houtaomuga site, dated 7 kya, carried N1a1a1a1a-M2117, which was genetically linked to early Neolithic (EN) Amur River Basin individuals (Ning et al. 2020a). Further analysis revealed that the IA Xinjiang individuals, BA West Liao River individuals, and several southern Siberian ancient individuals belonged to N1a or its sublineages (Fig. 2), which correlated significantly with ANA-related ancestral components (Fig. 5b). N1a2-F1008/L666 comprised approximately 67% of the N1a sublineages, bifurcating into N1a2a and N1a2b approximately 9.5 kya; N1a2a1, which made up the largest proportion of N1a2a (∼82%), which diversified approximately 4.4 kya; and N1a2b, which diverged approximately 4.0 kya (Fig. 1b; supplementary fig. S1, Supplementary Material online). Ancient DNA data revealed that EN Shamanka individuals from Cis-Baikal and several southern Siberian ancients belonged to N1a2a (Fig. 2). Early diffusion centers for N1a2 were identified in North China and the southeastern part of Northeast China (supplementary fig. S17b, Supplementary Material online). N1b-F2930 is primarily found in Tibeto-Burman-speaking populations in low-altitude Southwest China (∼24%) and less frequently in other Chinese populations (supplementary table S5, Supplementary Material online). Notably, ancient East Asians belonging to sublineage N1b, specifically N1b2 or its derivatives, are mainly distributed on the Tibetan Plateau (Fig. 2). To better understand the phylogeographic origins of N-M231 and its N1a/N1b subclades and the factors influencing their distribution patterns, further collection and whole Y-chromosome sequencing of spatiotemporally distinct ancient and modern Eurasians belonging to N sublineages are essential.

Haplogroup C-M130, one of the primary paternal lineages in East Asia and likely carried by early settlers, diverged approximately 50 kya (Fig. 1b). Its subclade C2-M217, which is particularly widespread in North China, exhibited a notable frequency across multiple regions (Figs. 2 and 3a and c; supplementary fig. S17b, Supplementary Material online). The earliest known individual carrying C2-M217, designated as AR19K, dates from 19,587 to 19,175 cal BP in the Amur River Basin (Fig. 2). Distinct patterns are observed for the C2a-L1373 and C2b-F1067 subclades. C2a-L1373, sometimes referred to as the “northern branch,” shows the highest frequencies in Inner Mongolia, whereas C2b-F1067, the “southern branch,” is most prevalent in Central, North, and Northeast China (Fig. 3c). The C2a subclade, particularly C2a1a, is predominant among Transeurasian-speaking populations, with C2a1a1b1-F1756, C2a1a2a-M86, and C2a1a3a-F3796 identified as major subclades within China (Fig. 2; supplementary table S5, Supplementary Material online). BEAST-based phylogenetic analysis revealed that C2a1a1b1 diversified into C2a1a1b1a and C2a1a1b1b approximately 5.4 kya (Fig. 1b; supplementary fig. S1, Supplementary Material online), which are widely found in the northern Han, Mongolic and Tungusic people (supplementary table S5, Supplementary Material online). Historical dispersal of these subclades is evidenced by their presence in BA West Liao River, IA Amur River Basin, and several BA to Historical Era (HE) individuals from the Mongolian Plateau (Fig. 2), suggesting links to early expansions of Mongolic/Tungusic ancestors. Furthermore, C2a1a2a sublineages are common in Transeurasian groups across East Asia and North Asia (Fig. 2; supplementary table S5, Supplementary Material online). A Mesolithic Amur River Basin individual (AR11K), an ANA-representative Boisman_MN, and two HE Mongolian Plateau individuals carried C2a1a2 or C2a1a2a (Fig. 2), indicating that migration from the Amur River Basin to the Mongolian Plateau contributed to the genetic makeup of the current Transeurasians, particularly Mongolic/Turkic speakers (supplementary table S5, Supplementary Material online). C2a1a3a-F3796, also known as the C2*-Star Cluster, diverged from C2a1a3 approximately 3.7 kya, predating previous estimates (Wei et al. 2018). This may be due to sampling bias and differences in TMRCA estimation methods based on Y-STRs and Y-chromosome sequences. This sublineage is foundational among Mongolic-speaking populations. One Neolithic Amur River Basin individual, one MN Boisman individual, several HE Mongolian Plateau ancients, and IA Xinjiang samples are classified under the C2a1a3 sublineages (Fig. 2). Additionally, C2a sublineages are also identified in central and southern Chinese populations (supplementary table S5, Supplementary Material online), suggesting their southward migration from North Asia, likely driven by the expansion of the Mongol Empire.

The phylogenetic analysis of C2b-F1067 indicated that ancient populations carrying its sublineages significantly enriched the gene pool of modern eastern Eurasians. Observations suggest that Inner Mongolia and Northeast China were likely initial dispersal centers for C2b, exhibiting distinct geographical distribution patterns (supplementary fig. S17b, Supplementary Material online). For instance, C2b1a1-CTS2657 is found at high frequencies in North and Northeast China, while C2b1a2-F3880 predominates in Northeast and North China, as well as in East China, notably in Shandong, Jiangsu, and Shanghai. Conversely, C2b1b-F845 had the highest frequencies in Central China, Southwest China (mainly Guizhou), and SEA (supplementary fig. S17b, Supplementary Material online). The distribution patterns identified for the C2b sublineages, which partly diverge from previous studies (Wu et al. 2020), may result from sampling biases and differences in reference populations. Our analysis confirmed the southern origin of C2b1b-F845 (supplementary fig. S17b, Supplementary Material online) and identified two ancient individuals from Shigatse on the Tibetan Plateau with C2b1 mutations, one late Neolithic (LN) Shimao individual belonging to C2b1a2b1, and only two Neolithic Yellow River Basin farmers and one HE Tibetan Plateau individual associated with C2b1b sublineages (Fig. 2). To comprehensively explore the phylogeographic origin and dispersal of C2b1 sublineages, further analysis of spatiotemporally diverse ancient southern East Asians (ASEA), particularly from low-altitude regions, is needed. Statistically significant negative correlations between pairwise Fst genetic distances and the frequency of western Eurasian/Siberian-related lineages underscore their contribution to the genetic differentiation between northern and southern East Asians (supplementary fig. S14b, Supplementary Material online). Overall, genetic analyses incorporating the haplogroup frequency spectra of modern and ancient East Asians revealed a robust genetic connection between the descendants of Neolithic southern Siberian hunter-gatherers and modern East Asians. The geographical distribution patterns and TMRCA estimates of C2a1a/C2b1a/N1a1/Q1a1a-derived sublineages support the hypothesis that ancient migrations of West Liao River millet farmers have shaped the current distribution patterns in Chinese populations, particularly among Transeurasian speakers. These findings align with earlier findings triangulated from linguistic, archaeological, and genetic evidence (Robbeets et al. 2021).

#### Traces of the Early Asian and Ancient Northern East Asian Millet Farmer-Related Lineages in China

The ancient genetic connections among Andamanese, Jomon-related indigenous Japanese, and highland Tibetans are evidenced by shared Paleolithic ancestral components and the uniparental D lineage. Analysis of the phylogeographic origins of D subclades revealed that D1-M174, a major paternal haplogroup in East Asians, is prevalent in our YHC (supplementary fig. S16, Supplementary Material online). Haplogroup D1a, which is particularly frequent in the Tibetan Plateau, is predominantly subdivided into D1a1a-M15 and D1a1b-P99, with these divisions occurring approximately 46 kya (Fig. 1b; supplementary fig. S1, Supplementary Material online). D1a1a sublineages are commonly found (>54%) among Tibeto-Burman-speaking populations in Southwest China and are less frequent in other Chinese populations, while D1a1b sublineages are most prevalent on the Tibetan Plateau (>36%, supplementary table S5, Supplementary Material online). D1a1a sublineages are frequently found in the Mongolian and Tibetan plateaus and Yellow River Basin ancients, and D1a1b sublineages are mainly found in the Tibetan Plateau ancients (Fig. 2). The distribution patterns of these sublineages in both modern and ancient East Asians provide direct evidence of their migration paths: D1a1a-M15 likely migrated northward through western Sichuan to the Gansu–Qinghai region and possibly into the Himalayan area along the Tibetan-Yi corridor; D1a1b-P99, particularly its subclade D1a1b1-P47, originated on the Tibetan Plateau. These D1a sublineages are predominantly found in Tibetan populations, supported by genetic contributions from northern Chinese millet farmers via a revised Y-chromosome phylogeny and correlations with O2 sublineages and Lubrak-related Tibetan Plateau ancestry (Fig. 4d; supplementary fig. S14c, Supplementary Material online). Gene flow events and the presence of Lubrak-related D sublineages significantly influenced the genetic diversity patterns. Notably, the frequencies of four lineages (O2a2b1, O2a2b1a, O2a2b1a1, and O2a2b1a1a) strongly correlate with the Lubrak-related ancestry, confirming that Neolithic expansions from the Yellow River Basin contributed to the peopling of the Tibetan Plateau (Fig. 5c). Ancient DNA evidence from autosomal variations and maternal lineages further underscores the substantial impact of Neolithic millet farmers on the permanent settlement of the Tibetan Plateau (Wang et al. 2023).

Archeological evidence indicates that millet-based agriculture independently emerged in the Yellow River Basin and West Liao River at approximately 6,000 BCE, fostering the development of foxtail (*Setaria italica*)-prevalent Yangshao and broomcorn (*Panicum miliaceum*)-prevalent Xinglongwa cultures, respectively (Miller et al. 2016; Leipe et al. 2019). Leipe et al. noted that shifts in agricultural practices from approximately 6000 to 2,000 BCE led to a quasi-exponential population growth in North China, aligning with the major dispersal of Sino-Tibetan-speaking populations from the Yellow River Basin during the fourth millennium BCE (Leipe et al. 2019). Ancient DNA analyses of millet farmers from the Yangshao and Longshan cultures suggested that the Sino-Tibetan people originated in North China (Ning et al. 2020b). The Haojiatai-related ancestry dominant in Chinese populations correlated strongly with the O/Q/C/N lineages (Figs. 4e and 5d). O-M175, which is prevalent in East and Southeast Asians, includes the significant O1-F265 and O2-M122 subclades, whose expansions are associated with the spread of millet and rice agriculture from domestication centers in the Yellow River Basin, West Liao River, and Yangtze River Basin (Fig. 5d to f). The influence of Ancient Northern East Asian (ANEA) on modern East Asian paternal genetic diversity requires a further comprehensive assessment. O-related sublineages, with O2 lineages diversifying approximately 29 kya (Fig. 1b), are broadly distributed in North China and the Tibetan Plateau (Figs. 2 and 3a). O2-M122, particularly subclade O2a-M324, is a major paternal lineage in East and Southeast Asians, showing a strong correlation in distribution patterns (Fig. 3d; supplementary figs. S17, and S18c, Supplementary Material online). O2a-M324 is found at high frequencies along China's coast and surrounding areas (>52%), suggesting ancestral migration routes along the coast extending into SEA (supplementary fig. S17b, Supplementary Material online). An ancient individual from the MN West Liao River Hongshan culture identified as belonging to O2a-M324 supports this lineage's association with early cultural developments in Northeast China. Systematic evidence further corroborated that O2a-M324 originated in Northeast China, particularly in Heilongjiang Province, where it remains highly prevalent (supplementary fig. S17b, Supplementary Material online). However, the high frequencies also observed in eastern coastal provinces like Shandong, Shanghai, Fujian, and Guangdong may reflect sampling biases and historical migrations, notably during the Chuangguandong movement. Additionally, the optimized hot spot analysis results suggest that the middle and lower reaches of the Yellow River Basin were early diffusion centers for O2a-M324 (supplementary fig. S17b, Supplementary Material online).

Distinct distribution patterns were observed for the O2a1-L127.1 and O2a2-JST021354/P201 sublineages (supplementary fig. S17, Supplementary Material online). O2a1 is most prevalent in Southeast China, with its frequency decreasing in adjacent regions (supplementary fig. S17c, Supplementary Material online). Most of the O2a1 subclades show similar distribution patterns (supplementary fig. S17c to d, Supplementary Material online). O2a2 has the highest frequency in the Tibetan Plateau, Southeast China, and SEA (supplementary fig. S17f, Supplementary Material online). The sublineage O2a2a-M188 is notably frequent in the SEA, decreasing in frequency from south to north across East Asia (supplementary fig. S17f, Supplementary Material online); O2a2b-P164 is widespread in China, with the highest occurrence on the Tibetan Plateau (supplementary fig. S17h, Supplementary Material online). The majority of O2a1 individuals (∼87%) are O2a1b-JST002611, which is widespread across Chinese populations, particularly among Han populations (supplementary fig. S17d and table S5, Supplementary Material online). However, O2a1b and its sublineages appear infrequently among Tibeto-Burman groups, suggesting a minimal impact on this population. The initial diffusion centers for O2a1b sublineages are identified in the middle and lower reaches of the Yellow River Basin (supplementary fig. S17d, Supplementary Material online). Two main sublineages, O2a1b1a1a1a-F11 and O2a1b1a2a-F238, are found with differing frequencies; O2a1b1a1a1a-F11 is more common, especially in diverse Han populations (supplementary table S5, Supplementary Material online). O2a1b1a1a1a expanded approximately 8.9 kya, and O2a1b1a2a diverged approximately 9.0 kya (Fig. 1b; supplementary fig. S1, Supplementary Material online), which are the times that greatly preceded earlier TMRCA estimates. This discrepancy highlights differences between the Y-STR and Y-SNP-based TMRCA estimations and the influence of the Y-chromosome sequence coverage. O2a1b1a1a, the upstream lineage of O2a1b1a1a1a, appears most frequently in Southeast China and Guizhou in Southwest China, and its initial diffusion center is likely to be the middle and lower portions of the Yellow River Basin (supplementary fig. S17e, Supplementary Material online). O2a1b1a1a1a-F11 was identified in a Banpo site sample (Zhang et al. 2018), linking its emergence to Yangshao millet farmers. Furthermore, historical individuals from Shigatse on the southern Tibetan Plateau and Mongolian Plateau also carried this sublineage (Fig. 2), indicating the significant influence of Neolithic millet farmers. The O2a1b lineage was also detected in the 500-year-old GaoHuaHua (Fig. 2), establishing a connection with Yangshao millet and ASEA rice farmers (Miao et al. 2021). Linguistic evidence points to the initial divergence of Sino-Tibetan languages during the Yangshao period, with their dispersal likely occurring in the upper Yellow River Basin (Zhang et al. 2019). The estimated expansion of O2a1b1a1a1a and the divergence of Sino-Tibetan languages, in addition to paleogenomic evidence, suggest significant genetic contributions from ANEA millet farmers to modern Sino-Tibetan groups in China. Notably, the diffusion center for Sino-Tibetan-related ancestors with O2a1b1a1a1a does not align with the dispersal center of Sino-Tibetan languages, highlighting potential discrepancies due to real differences, sampling bias, or limitations in computational biology algorithms. Thus, further extensive sampling of modern and ancient East Asians is recommended to refine these findings.

High frequencies of most O2a2a subclades are observed in South China and SEA (supplementary fig. S17f and g, Supplementary Material online). Among these sublineages, the O2a2a1a2-M7 sublineages constitute the largest proportion (∼43.8%, supplementary table S5, Supplementary Material online) and are primarily found in the Hmong-Mien people and southern Han Chinese (supplementary table S5, Supplementary Material online). Only one IA Hanben individual from Taiwan Island was identified within O2a2a1a2a2 (Fig. 2). A recent rapid expansion of O2a2a1a2a1a1a2a1a1a1 around 2.9 kya was noted (Fig. 1b). Moreover, the O2a2b sublineages are widely distributed across China (supplementary fig. S17h and table S5, Supplementary Material online). O2a2b1-M134, a major subclade of O2a2b, appears predominantly among Sino-Tibetan speakers (∼85%), with the highest occurrence in the Tibetan Plateau (supplementary fig. S17h and table S5, Supplementary Material online). Two star-like expansions have been linked to O2a2b1a1a1-F8 (∼7.3 kya) and O2a2b1a2a1a-F46 (∼9 kya) (Fig. 1b; supplementary fig. S1, Supplementary Material online). The upstream lineage of O2a2b1a1a1, O2a2b1a1a, is prevalent in Southwest/Southeast China and the Circum-Bohai-Sea region (supplementary fig. S17h, Supplementary Material online). The frequencies of O2a2b1a2 and the upstream lineage of O2a2b1a2a1a are greater in Northeast, North, and East China than in other areas (supplementary fig. S17h, Supplementary Material online). The optimized hot spot analysis suggests that the early diffusion center for O2a2b1a2 is likely the Circum-Bohai-Sea region (supplementary fig. S17h, Supplementary Material online). Several LN to IA ANEA millet farmers, HE Mongolian Plateau ancients, IA to HE Xinjiang individuals, and multiple IA to HE Tibetan Plateau individuals, particularly those in the southern Tibetan Plateau, are assigned to the sublineages of O2a2b1a1. Additionally, some ancient individuals from the Yellow River Basin and Northeast/Southeast Tibetan Plateau are linked to O2a2b1a2a1a or its sublineages (Fig. 2). Star-like expansions noted in O2a1b1a1a1a-F11 (∼8.9 kya), O2a2b1a1a1-F8 (∼7.3 kya), and O2a2b1a2a1a-F46 (∼9 kya) represent approximately 27% of the newly reported paternal lineages and 31% of the paternal lineages in China (supplementary fig. S1 and tables S4 and S5, Supplementary Material online), highlighting significant contributions from the Neolithic expansions of ANEA millet farmers to modern Chinese gene pools. Consequently, the development of millet agriculture, migration of ancient millet farmers, and admixture with diverse indigenous populations have shaped the present distribution of the O2a-M324 sublineages, particularly O2a1b-JST002611 and O2a2b1-M134.

#### ASEA Rice Farmer-Related Founding Lineages from Yangtze River Basin Left a Massive Genetic Legacy in China and SEA

Southern East Asia, an origin center for rice domestication, is considered the ancestral homeland of the Hmong-Mien, Tai-Kadai, Austroasiatic, and Austronesian people. ADMIXTURE models suggested that Hmong/Hanben-related ancestral components prevalent in southern Chinese populations are associated with most O1 subclades (Figs. 4f and g and 5e). Recent studies have shown that ancient Yangtze River Basin rice farmers influenced the genetic makeup of ancient Yellow River Basin millet farmers and populations in SEA and Oceania (Yang et al. 2020; Wang et al. 2021a). The exploration of the phylogeographic features of the O1 sublineages revealed a high prevalence of O1-F265 across Southeast, South, and Southwest China, SEA, and the Japanese archipelago. The subclade O1a-M119 is common in Southeast China, while O1b-M268 predominates in Southwest China and SEA (supplementary fig. S18 and table S5, Supplementary Material online). The O1a sublineages are primarily found among Austronesian-, Tai-Kadai-, and Sinitic-speaking populations in Southeast, South, and Southwest China (supplementary table S5, Supplementary Material online), suggesting a shared patrilineal origin among these groups and a significant gene flow with the Han Chinese (Chen et al. 2022; Wang et al. 2022; Liu et al. 2023). A Neolithic expansion linked to subclade O1a1a1 (∼7.6 kya, supplementary fig. S1, Supplementary Material online) is identified, with O1a1a1a being more prevalent than O1a1a1b (supplementary fig. S18b and table S5, Supplementary Material online). O1a1a1a and its sublineages, which are found predominantly in Southeast China (∼51%), diversified approximately 5.7 kya, with early dispersal centers likely in the middle and lower portions of the Yangtze River Basin and the southeast coast (supplementary fig. S18b, Supplementary Material online). O1a1a1b, which has the highest frequency in Hainan among the Li people, decreases from south to north, with initial dispersal centers in South/Southwest China. This lineage, which is possibly ancestral to the Baiyue, significantly contributed to other Chinese populations (supplementary fig. S18b and table S5, Supplementary Material online). Another Neolithic expansion associated with O1a1a2 (∼8.4 kya, supplementary fig. S1, Supplementary Material online) shows high frequencies along the southeastern coast, South/Southwest China, and Vietnam, with likely initial dispersal centers in South/Southwest China (supplementary fig. S18b, Supplementary Material online). The primary sublineage O1a1a2a diverged approximately 6.4 kya (supplementary fig. S1, Supplementary Material online). The geographical distribution patterns and divergence times of O1a1a1b- and O1a1a2a-related lineages align with inferred migration routes from coastal to inland Southwest China and from Southwest China to mainland SEA according to the phylogenetic reconstructions of the Tai-Kadai languages (Tao et al. 2023). Several Taiwanese Hanben individuals are found within O1a1a1a1 sublineages (Fig. 2), and evidence from the Liangzhu culture in the Yangtze River Delta indicates that rice farmers carrying O1a-M119 in the Yangtze River Basin were likely the direct ancestors of the modern Tai-Kadai and Austronesian people, profoundly influencing southern Han Chinese. This migration proceeded southward along China's southeastern coast or inland routes to Southeast/Southwest China and mainland SEA.

Haplogroup O1b-M268, predominantly found in Southwest/South China, SEA, and the Japanese archipelago, is divided into three major subclades, namely, O1b1a1-PK4, O1b1a2-Page59, and O1b2-P49, each displaying distinct distribution patterns (supplementary fig. S18d to f, Supplementary Material online). O1b1a1 and its sublineages, which are mainly located in Southwest/South China and SEA, constitute key paternal lineages among the Tai-Kadai-speaking populations (supplementary table S5, Supplementary Material online). However, O1b1a1a-M95, primarily found in Austroasiatic groups, suggests an ancient gene flow between the proto-Austroasiatic and proto-Tai-Kadai populations, highlighting the impact of limited Austroasiatic sample sizes in our data set (Zhang et al. 2014; Kutanan et al. 2019; Macholdt et al. 2020). Ancient DNA analysis revealed that individuals from ∼3,000 years ago at the Wucheng site in Jiangsu along the Yangtze River Basin and from the Hengbei site in Shanxi, as well as several ∼1,500-year-old Guangxi individuals from southern East Asia (Li et al. 2007; Zhao et al. 2014), carried O1b1a1a-M95 or related sublineages (Fig. 2). Additionally, recent expansion events associated with O1b1a1a1a1b1a1a1 (∼3 kya) and O1b1a1a1a1b2a1a1a (∼2.5 kya) have been identified. O1b1a2 and its sublineages, which are relatively rare in East Asia, are primarily found in East China, the southeastern part of Northeast China, and Vietnam, especially among Han Chinese individuals (supplementary fig. S18e and table S5, Supplementary Material online). An MN individual from the Wanggou site, which is part of the Yangshao culture, was identified as belonging to O1b1a2-Page59 (Fig. 2). O1b2-P49 is most frequent in Japan, followed by Northeast China, but its detailed phylogenetic structure has yet to be fully elucidated (supplementary fig. S18f, Supplementary Material online). The genetic diversity patterns of the O1 lineages indicate a significant influence of ancient rice farmers on the gene pools of populations in South China and SEA. The complex movements and admixture events associated with these ancient agriculturists have profoundly shaped the genetic landscape of modern and ancient East Asians. To clarify the origins of crucial Chinese-dominant subclades and the demographic processes influencing modern Chinese populations, we should design a systematic sampling strategy. This approach should include comprehensive Y-chromosome sequences and the collection of spatiotemporally distinct ancient samples for more detailed analyses.

## Conclusion

Genetic evidence from autosomal DNA studies has profoundly transformed our understanding of the genetic histories of diverse human populations. However, research into the ancient genetic legacy reflected in modern Chinese populations via Y-chromosome analysis remains sparse. To address this gap, we used the YHC to analyze the Y-chromosome diversity in ethnolinguistically diverse Chinese populations through whole Y-chromosome sequencing and our newly developed high-resolution YHSeqY3000 panel. This project reconstructs demographic events, such as isolation, expansion, and admixture, using various computational models. The new data were integrated with a Y-chromosome genomic database of 14,644 individuals, creating a comprehensive database that includes 1,786 ancient Eurasians and 115 modern Chinese populations from 47 ethnic groups. This integration facilitates an in-depth exploration of the paternal genetic diversity of Chinese populations. Our findings indicate that multiple founding lineages associated with millet/rice farmers from the Yellow River Basin and the Yangtze River Basin, Siberian hunter-gatherers, and ancient western Eurasian pastoralists and farmers significantly influence the geographical patterns of paternal genetic stratification in Chinese populations. There is a strong correlation between the frequency of subsistence model-related founding lineages and the proportion of autosomal-based admixture from presumed ancestral sources, as well as between the latitude and a differentiated north-to-south genetic matrix. These correlations suggest that ancient migrations and extensive admixtures with indigenous populations primarily shaped the paternal genetic landscape of Chinese populations. To further elucidate the paternal evolutionary history of East Asians, we emphasize the importance of combining high-depth whole-genome sequencing data from both modern and spatiotemporally diverse ancient populations. This comprehensive approach will enhance our understanding of the dynamic interplay between migration, admixture, and cultural development in this region.

## Materials and Methods

### Sampling, Sequencing, Genotyping, and Phylogenetic Construction

#### Study Participants

To comprehensively characterize the paternal diversity across China, saliva samples were collected from 919 participants representing 39 ethnolinguistic groups (supplementary table S1, Supplementary Material online). The participants were all descendants of self-identified ethnic group members, with their grandparents having resided in their respective sampling districts for at least three generations. The study received approval from the Medical Ethics Committee of West China Hospital of Sichuan University (2023-306) and was conducted following the Helsinki Declaration of 2013 (World Medical Association 2013). Informed consent was obtained from each participant before sample collection.

#### DNA Extraction, Whole-Genome Sequencing, and Genotyping

Genomic DNA was extracted using the QIAamp DNA Mini Kit (QIAGEN, Germany). DNA concentrations were quantified with the Qubit dsDNA HS Assay Kit, following the standard protocol on a Qubit 3.0 fluorometer (Thermo Fisher Scientific). Sequencing was conducted on the Illumina platform (Illumina, San Diego, CA, USA), achieving 80× genome-wide coverage. The raw sequencing reads were mapped to the human reference genome GRCh37 using BWA v0.7.13 (Li and Durbin 2009). Duplicate reads were removed with Picard v3.0.0, followed by a base quality score recalibration via GATK v4.2.6.1. Joint variant calling was executed using GATK HaplotypeCaller, CombineGVCFs, and GenotypeGVCFs modules (McKenna et al. 2010). High-quality variant calls within a 10 Mb region were obtained through a sequence mask (Poznik et al. 2013). Variants exhibiting missing call rates greater than 5%, base quality below 20, and heterogeneity rate above 15% were filtered out using BCFtools v1.8 (Li 2011). Samples with missing call rates exceeding 5% were removed via vcftools v0.1.16 (Danecek et al. 2011). Ultimately, 914 samples meeting quality standards were selected for the downstream analysis, including the reconstruction of a time-scaled phylogenetic tree. Additionally, Y-specific target sequences with 100× coverage were generated using the custom-designed YHSeqY3000 panel on the MGI sequencing platform to validate the sequencing performance.

#### Haplogroup Classification and Phylogenetic Relationship Construction

The initial classification of the Y-chromosome haplogroups was performed using in-house scripts based on a newly reconstructed phylogenetic tree supplemented by classifications from HaploGrouper (Jagadeesan et al. 2021) and Y-LineageTracker (Chen et al. 2021), referencing the Y-DNA Haplogroup Tree 2019–2020 (https://isogg.org/tree/index.html). BEAST v1.10.4 (Suchard et al. 2018) facilitated the construction of a phylogenetic tree and the estimation of the TMRCA for various nodes using approximately 10 Mb of Y-chromosome sequences. B-related haplotypes served as an outgroup (Mallick et al. 2016). The optimal substitution model was selected via jModelTest v2.1.10 (Darriba et al. 2012). Markov chain Monte Carlo sampling was executed over 100 million iterations, with samples logged every 1,000 iterations and the initial 10 million iterations discarded as a burn-in. An exponential growth coalescent tree prior was used alongside the GTR (general time reversible) substitution model and a strict molecular clock. The substitution rate was set at 7.6 × 10^−10^ mutations per base pair per year (95% confidence interval: 6.7 × 10^−10^ to 8.6 × 10^−10^), as estimated by Fu et al. (2014). Three independent runs were amalgamated using LogCombiner, with the quality of the combined output manually verified using Tracer v1.7.1 (Rambaut et al. 2018). The maximum clade credibility tree was then generated with TreeAnnotator v1.10 and visualized using FigTree. To further investigate the ancient influences on the paternal landscape of the recently genotyped Chinese ethnic minorities, an ML phylogenetic tree was constructed using RAxML (Stamatakis 2014) with 914 ∼10 Mb of Y-chromosome sequences. Ancient genomes were integrated into this modern ML phylogeny using pathPhynder (Martiniano et al. 2022), and the tree was refined with iTOL (Letunic and Bork 2021). For the complete data set of Y-chromosome target sequences from 919 samples, a network-based analysis of shared haplotypes was conducted using PopART (Leigh and Bryant 2015), providing a comprehensive view of haplogroup relationships.

### Haplogroup Frequency Spectra Estimation and Clustering Analysis

#### Data Set Composition

We integrated previously published haplogroup data from 11,979 East Asian individuals across 79 populations drawn from key studies, the 1KGP, and the Human Genome Diversity Project (Poznik et al. 2016; Bergstrom et al. 2020). Additionally, data from 879 individuals across 27 SEA populations; 252 ancient East Asians from regions, including the Tibetan Plateau, Xinjiang, Amur River Basin, Yellow River Basin, West Liao River, and South China; and 1,534 ancient western Eurasians from the Allen Ancient DNA Resource were included (supplementary tables S2 and S3, Supplementary Material online; Mallick et al. 2024). A total of 13,777 modern individuals from 12 linguistically distinct groups were sampled, spanning 22 provinces, five autonomous regions, and four municipalities in China, as well as Thailand and Vietnam. These included 135 Austroasiatic-, 693 Austronesian-, 285 Hmong-Mien-, 75 Japonic-, 35 Koreanic-, 994 Mongolic-, 863 Tai-Kadai-, 1338 Tibeto-Burman-, 260 Tungusic-,1 Indo-European-, 291 Turkic-, and 805 Sinitic-speaking Hui, 3,248 northern Han Chinese, and 4,754 southern Han individuals (supplementary tables S1 and S2, Supplementary Material online). The haplogroups were manually revised according to variant information and the Y-DNA Haplogroup Tree 2019–2020. To facilitate the estimation of the spatial distributions of the paternal lineages, we aggregated haplogroup data to create metapopulations based on geographical region, ethnicity, and language family. The haplogroup frequencies were estimated at various levels of terminal haplogroups. Population genetic analyses were conducted on individual populations with sample sizes exceeding 10 and metapopulations exceeding 30.

#### Population Structure Inference

Pairwise Fst genetic distances were calculated from the haplogroup frequency spectra using Y-LineageTracker. MDS analyses were conducted based on these genetic distances utilizing the “cmdscale” function in R (https://itol.embl.de/itol.cgi). Additionally, PCA was performed on the haplogroup frequency spectra using Y-LineageTracker.

#### Spatial Statistics Correlated with the Phylogeographic Origin of Founding Lineages

The frequency of specific haplogroups within a province-defined population at various terminal haplogroup levels was computed using Y-LineageTracker, with level parameters adjusted from 0 to 6. The Chinese populations were grouped according to provincial administrative boundaries, while populations from the island and mainland SEA were aggregated by country. The spatial distribution patterns of the dominant haplogroups in China were examined using ArcMap. This included the application of the Getis-Ord General G method for optimized hot spot analysis and spatial autocorrelation analysis using Moran's I. The clusters identified through optimized hot spot analysis, referred to as hot and cold spots, approximated the potential geographical origins or diffusion centers of specific haplogroups, and the mirroring regions illustrated the general distribution trends of these haplogroups.

#### Autosomal-Based ADMIXTURE Estimation

A data set was constructed from 445 ancient individuals across 88 Eurasian populations and 1,325 modern individuals from 62 geographically diverse populations, sourced from our integrated 10K_CPGDP database. Admixture proportions of Chinese populations were estimated using model-based ADMIXTURE. The autosomal data set was pruned using PLINK (Chang et al. 2015) with the parameters “--indep-pairwise 200 25 0.4” and “--allow-no-sex”. Subsequently, ADMIXTURE was run with predefined ancestral sources ranging from 2 to 15 (Alexander et al. 2009). The optimal admixture model was determined based on the lowest cross-validation error values, and correlations between the haplogroup frequencies and autosomal-based admixture proportions of modern Chinese populations were estimated.

#### Correlation between Haplogroup Frequency and ADMIXTURE-Based Ancestral Proportion

The haplogroup frequencies of geographically defined metapopulations were initially calculated. The Chinese populations distinguished by geographic and ethnolinguistic characteristics were grouped by provincial administrative region. All examined lineages were truncated at the ninth level, identifying 139 common lineages with a frequency exceeding 0.05 in at least one population, 177 low-frequency lineages, and 165 rare lineages. Pearson's correlation coefficients between haplogroup frequencies and geographic coordinates (longitude and latitude), along with their intercorrelations and statistical significance, were estimated using the “corrplot” R package. Subsequently, all Chinese populations were consolidated into a single subpopulation, defining common lineages with frequencies above 0.01 or 0.05. The “corrplot” R package was also utilized to assess the correlation between admixture proportions and haplogroup frequencies.

## Declarations

### Ethics Approval and Consent to Participate

This study received approval from the Medical Ethics Committee of West China Hospital of Sichuan University and was conducted following the principles outlined in the Helsinki Declaration.

### Consent for Publication

Not applicable.

## Supplementary Material

msae122_Supplementary_Data

## Data Availability

All haplogroup information is provided in the Supplementary material. We followed the regulations of the Ministry of Science and Technology of the People's Republic of China. The raw genotype data required controlled access. Further requests for access to the raw data can be sent to Guanglin He (Guanglinhescu@163.com) and Mengge Wang (Menggewang2021@163.com).

## Appendix

### Full Author Lists of the 10K_CPGDP Consortium

Guanglin He^1^, Chao Liu^2^, Mengge Wang^2^, Renkuan Tang^3^, Libing Yun^4^, Junbao Yang^5^, Chuan-Chao Wang^6^, Jiangwei Yan^7^, Bofeng Zhu^8^, Liping Hu^9^, Shengjie Nie^9^, Hongbing Yao^10^

^1^Institute of Rare Diseases, West China Hospital of Sichuan University, Sichuan University, Chengdu, 610000, China

^2^Anti-Drug Technology Center of Guangdong Province, Guangzhou, 510220, China

^3^Department of Forensic Medicine, College of Basic Medicine, Chongqing Medical University, Chongqing, 400331, China

^4^West China School of Basic Science & Forensic Medicine, Sichuan University, Chengdu, 610041, China

^5^School of Basic Medicine and Forensic Medicine, North Sichuan Medical College, Nanchong, Sichuan, 637007, China

^6^State Key Laboratory of Cellular Stress Biology, School of Life Sciences, Xiamen University, Xiamen, 361005, China

^7^School of Forensic Medicine, Shanxi Medical University, Jinzhong, 030001, China

^8^Guangzhou Key Laboratory of Forensic Multi-Omics for Precision Identification, School of Forensic Medicine, Southern Medical University, Guangzhou, 510220, China

^9^School of Forensic Medicine, Kunming Medical University, Kunming, 650500, China

^10^Belt and Road Research Center for Forensic Molecular Anthropology, Gansu University of Political Science and Law, Lanzhou, 730000, China
