# Supplementary material for: Multiple Human Population Movements and Cultural Dispersal Events Shaped the Landscape of Chinese Paternal Heritage
Source: Mol Biol Evol. 2024 Jun 17;41(7):msae122. doi: 10.1093/molbev/msae122 (PMC11232699; doi:10.1093/molbev/msae122)
Supplement: msae122_Supplementary_Data [file msae122_supplementary_data.zip › Supplementary Meterials.pdf]

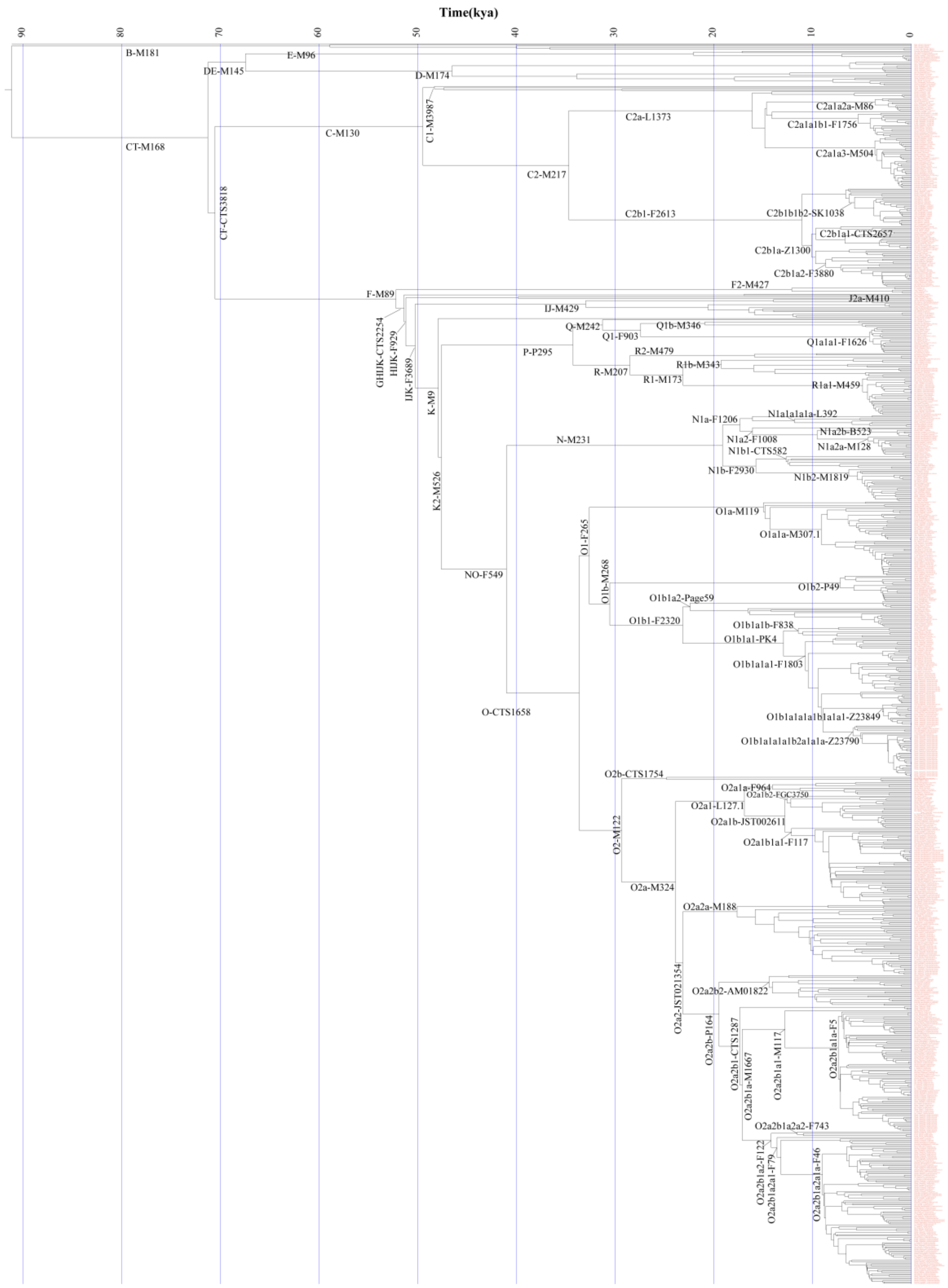

Fig. S1. The time-stamped Y-chromosome phylogenetic tree of 914 Chinese ethnic minority individuals. The B representative haplotypes from the Simons Genome Diversity Project were used as an outgroup. The branch length is proportional to the estimated time to the most recent common ancestor.

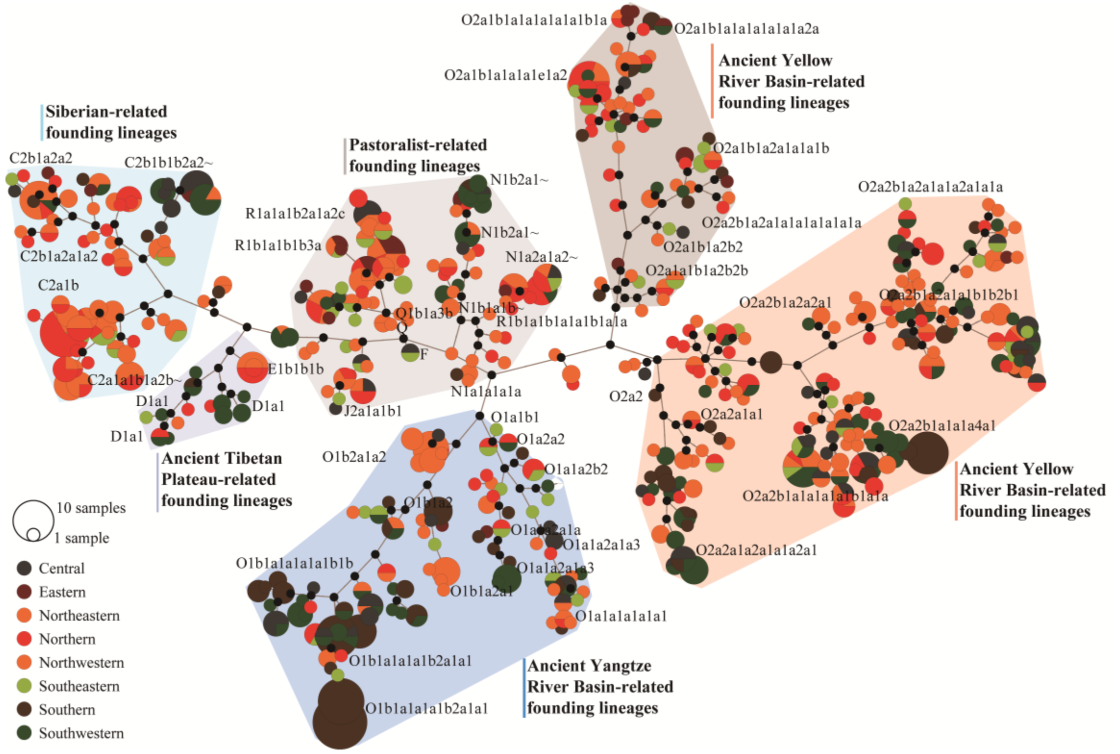

Fig. S2. Network relationships among 919 newly genotyped haplotypes were inferred based on the median-joining network algorithms. The target sequences were obtained based on the YHSeqY3000 panel. Different colors of the circle showed the geographical origin of one focused haplogroup, and different branches were labeled with their main paternal lineages and their possible related ancestral East Asians.

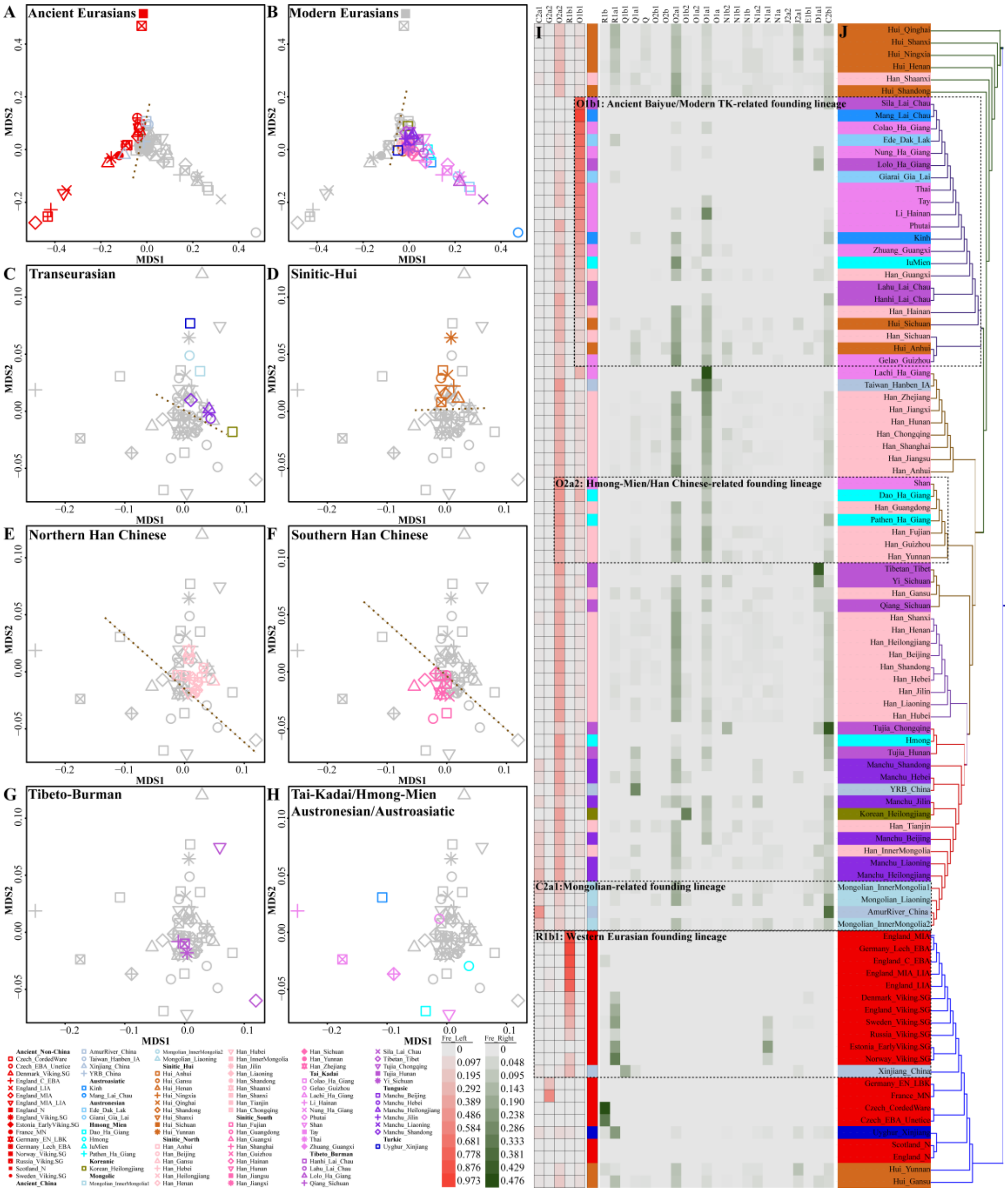

Fig. S3. Genetic diversity and population relationship patterns inferred from 12,606 individuals from 94 Eurasian populations.

(**A-B**) Multidimensional scaling analysis (MDS) based on the top two components showed the apparent genetic differentiation between Western Eurasians and modern East Asians. 601 ancient individuals from 21 Eurasian populations were collected from one curated compendium of ancient human genomes of the Allen Ancient DNA Resource (AADR). Modern populations included 87 Austroasiatic people from two populations, 51 Austronesian individuals from two populations, 231 Hmong-Mien people from four populations, 19 Koreans, 889 Hui individuals from 13 populations, 7367 Han Chinese individuals from 22 populations, 762 Tai-Kadai-speaking individuals from ten populations, 1161 Tibeto-Burman individuals from ten populations and 1438 Transeurasian individuals from nine populations.

(**C-H**) MDS results among 12,137 modern and ancient East Asian individuals from 77 populations, including 131 ancient individuals from populations in China, mainly collected from Xinjiang, Amur River Basin, Yellow River Basin and Southeast coastal regions.

(**I-J**) Frequency of major Y-chromosome lineages and phylogenetic relationships reconstructed based on the Fst matrix.

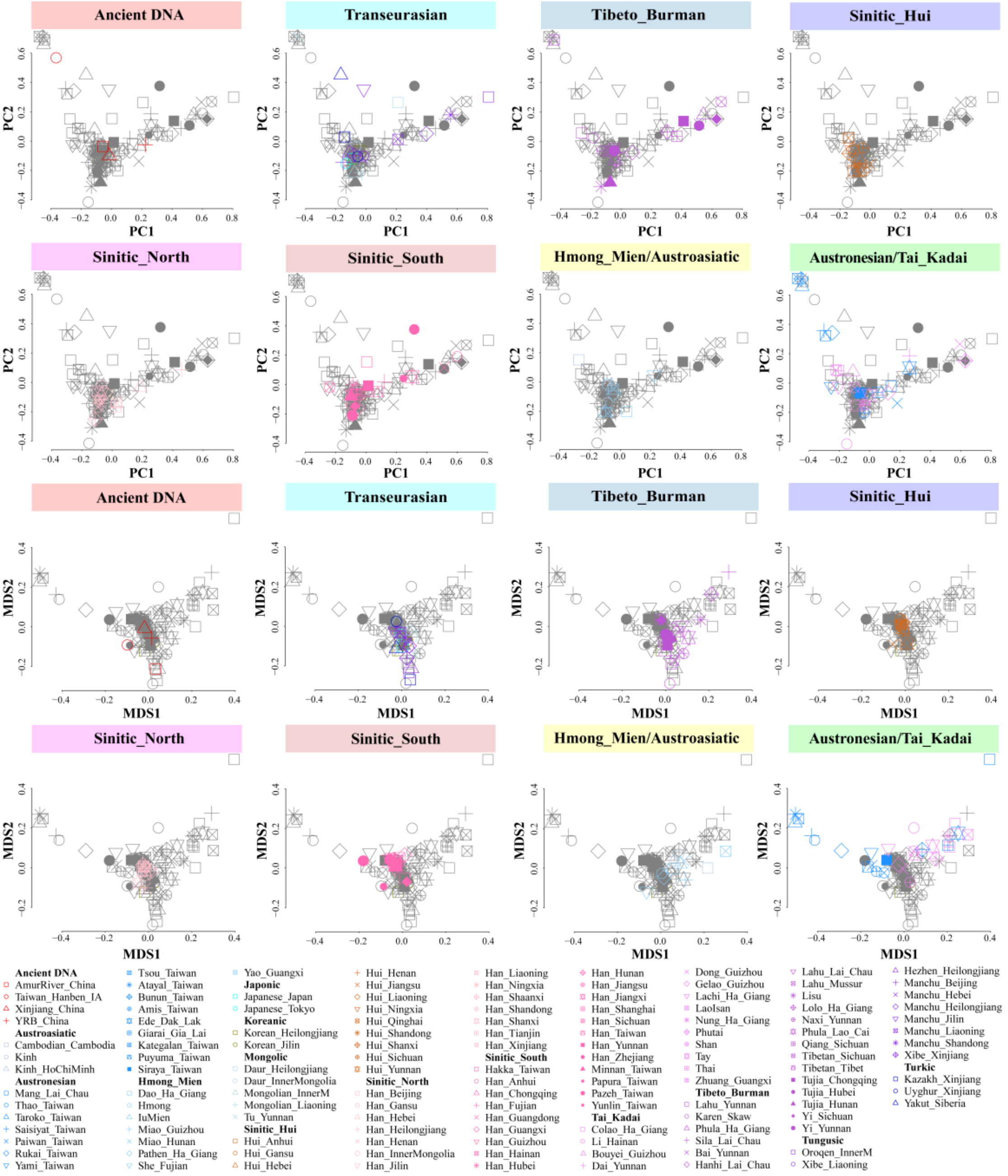

Fig. S4. Population genetic structure among 130 modern and four ancient East Asians. Principal Component Analysis and MDS based on the top two components showed the genetic similarities and differences between 134 populations, including 13,886 individuals.

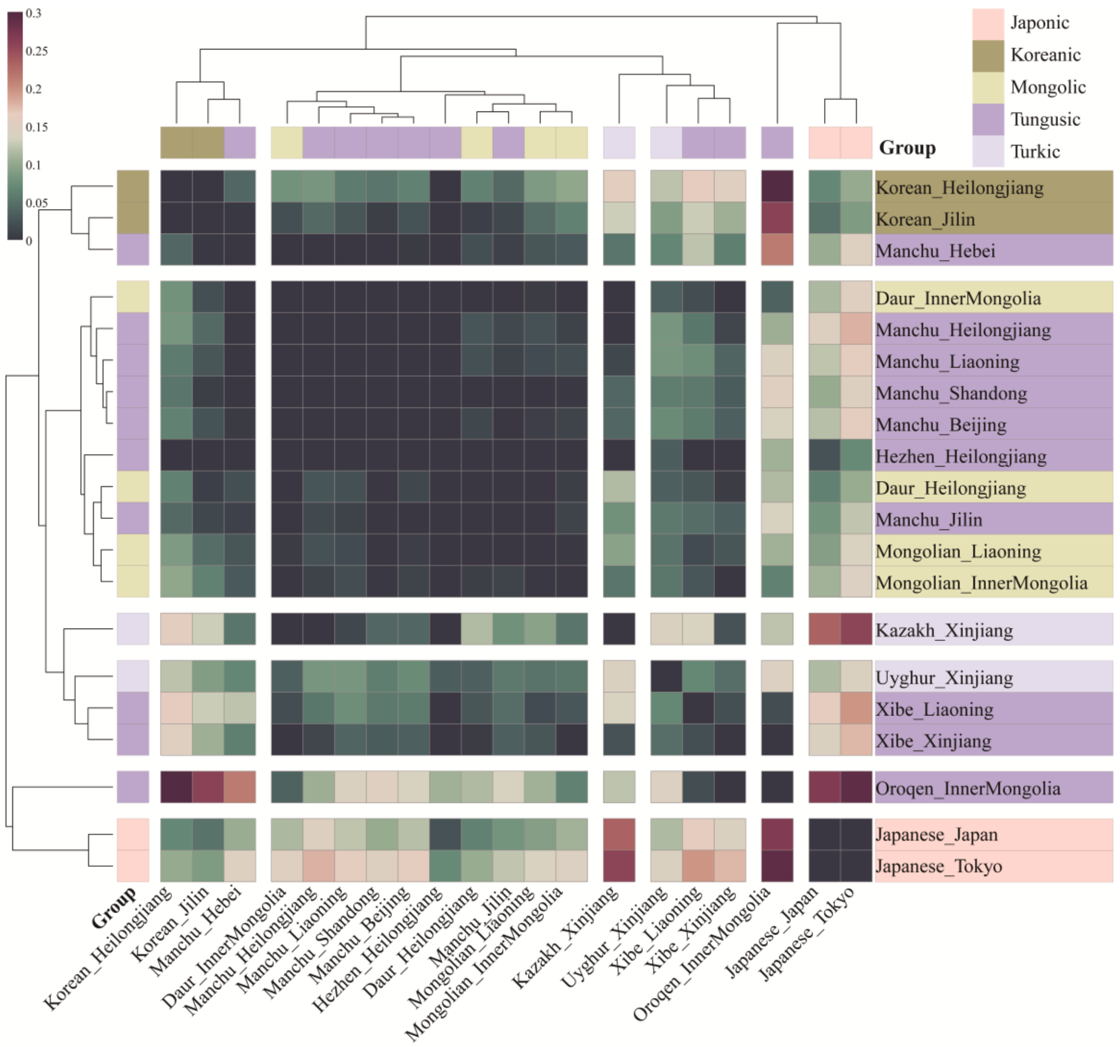

Fig. S5. Heatmap showed genetic affinity among Transeurasian populations.

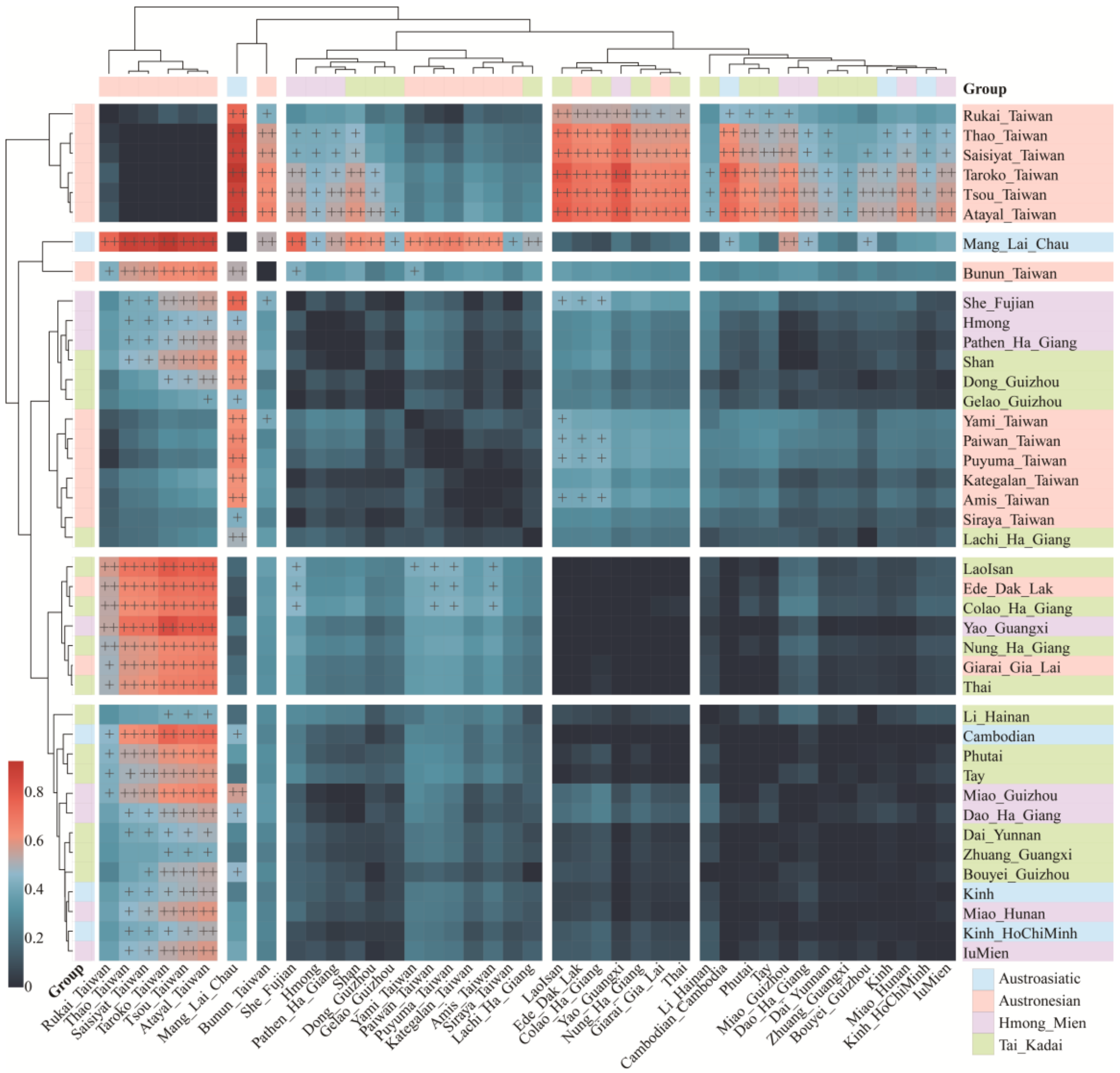

Fig. S6. Heatmap showed genetic affinity among Southern Chinese indigenous and Southeast Asian populations.

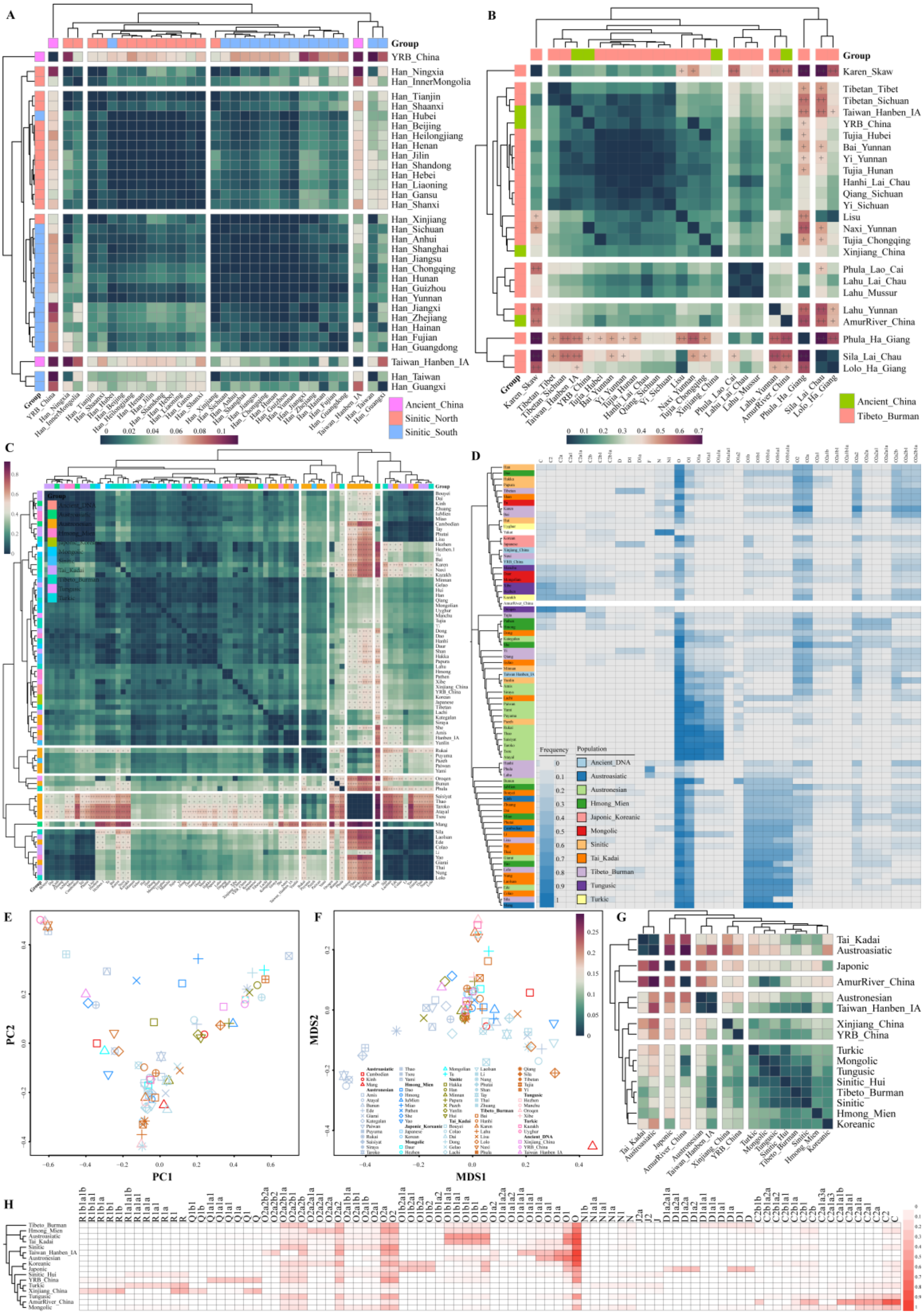

Fig. S7. Population genetic structure among modern and four ancient East Asians inferred from the haplogroup frequency spectrum (HFS).

(**A-B**) Heatmap showed genetic affinity among individual Sino-Tibetan-speaking populations. (**C-D**) Heatmap of Fst genetic matrix, phylogenetic relationship and the allele frequency of meta-ethnic groups. (**E-H**) Results of principal component analysis, multidimensional scaling plots, heatmap of the genetic distance matrix, phylogenetic relationship and HFS of Y-chromosome lineages.

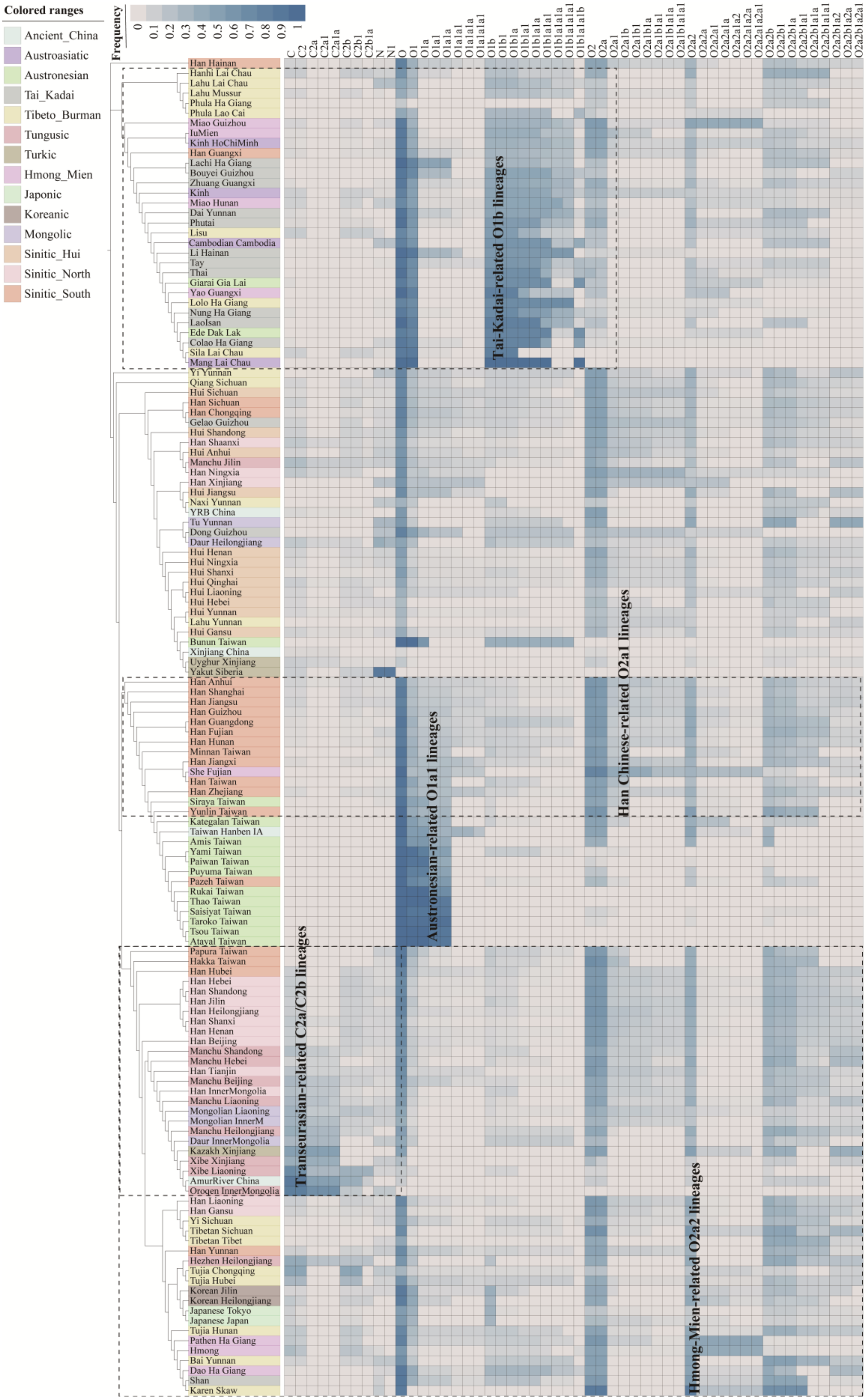

Fig. S8. **Phylogenetic relationships reconstructed based on the Fst matrix and frequency distribution of major Y-chromosome lineages.**

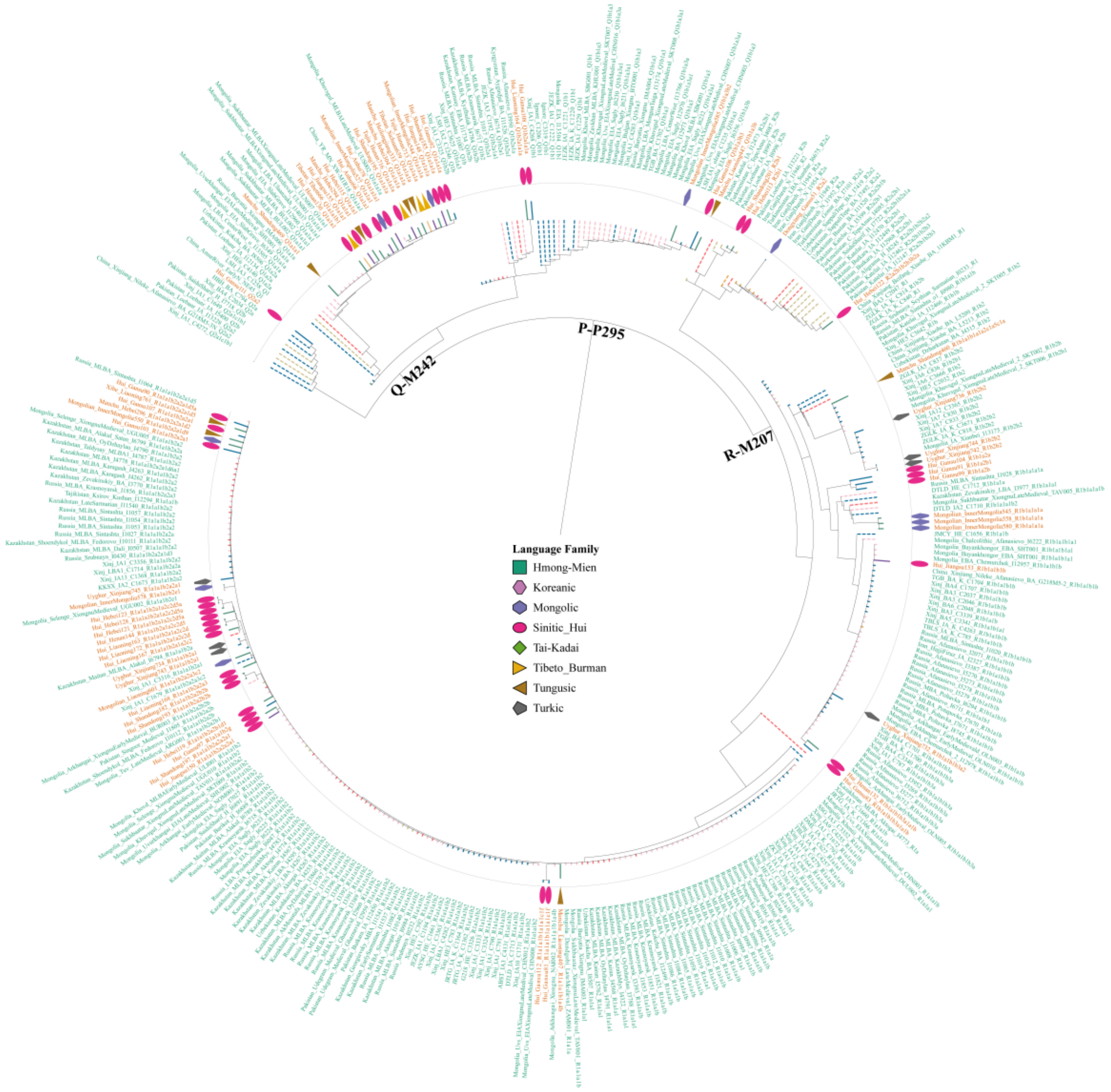

Fig. S9. The detailed Q/R-related maximum likelihood (ML) phylogenetic tree reconstructed among modern and ancient Eurasian populations. The legend is the same as shown in **Fig. 2**.

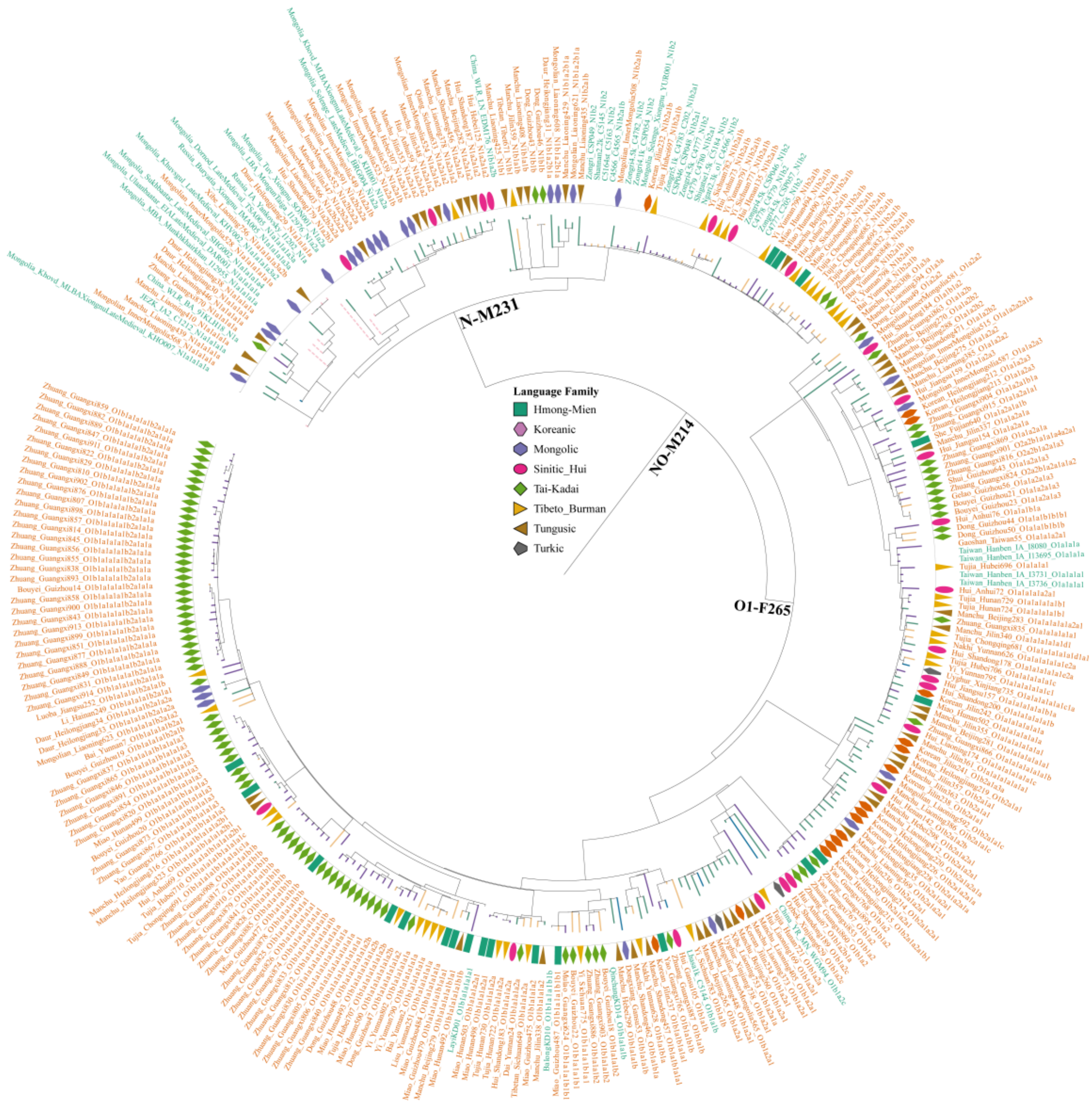

Fig. S10. The detailed N/O1-related maximum likelihood (ML) phylogenetic tree reconstructed among modern and ancient Eurasian populations. The legend is the same as shown in **Fig. 2**.

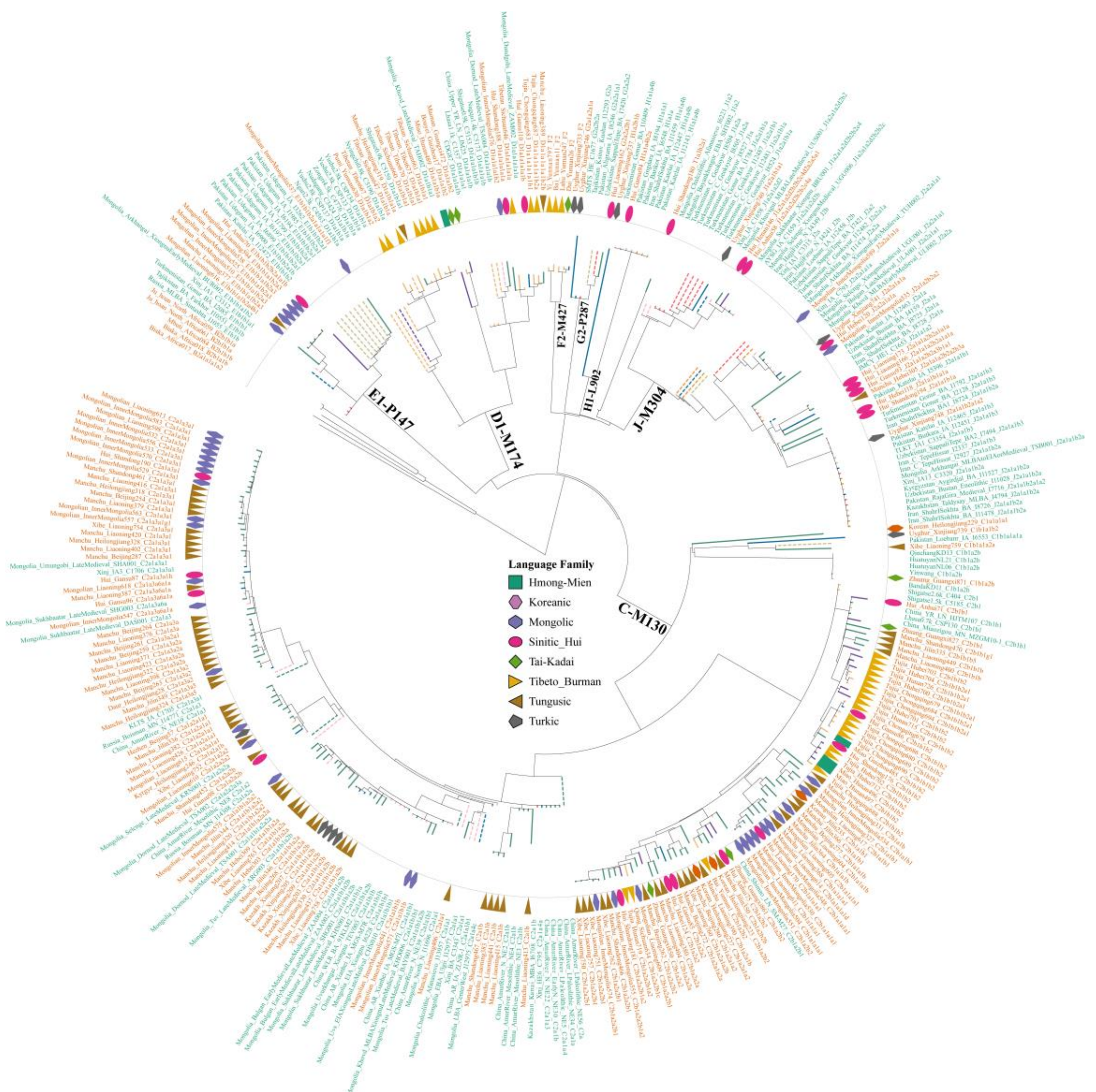

Fig. S11. The detailed E/D/F/C/J/G/H-related maximum likelihood (ML) phylogenetic tree reconstructed among modern and ancient Eurasian populations. The legend is the same as shown in **Fig. 2**.

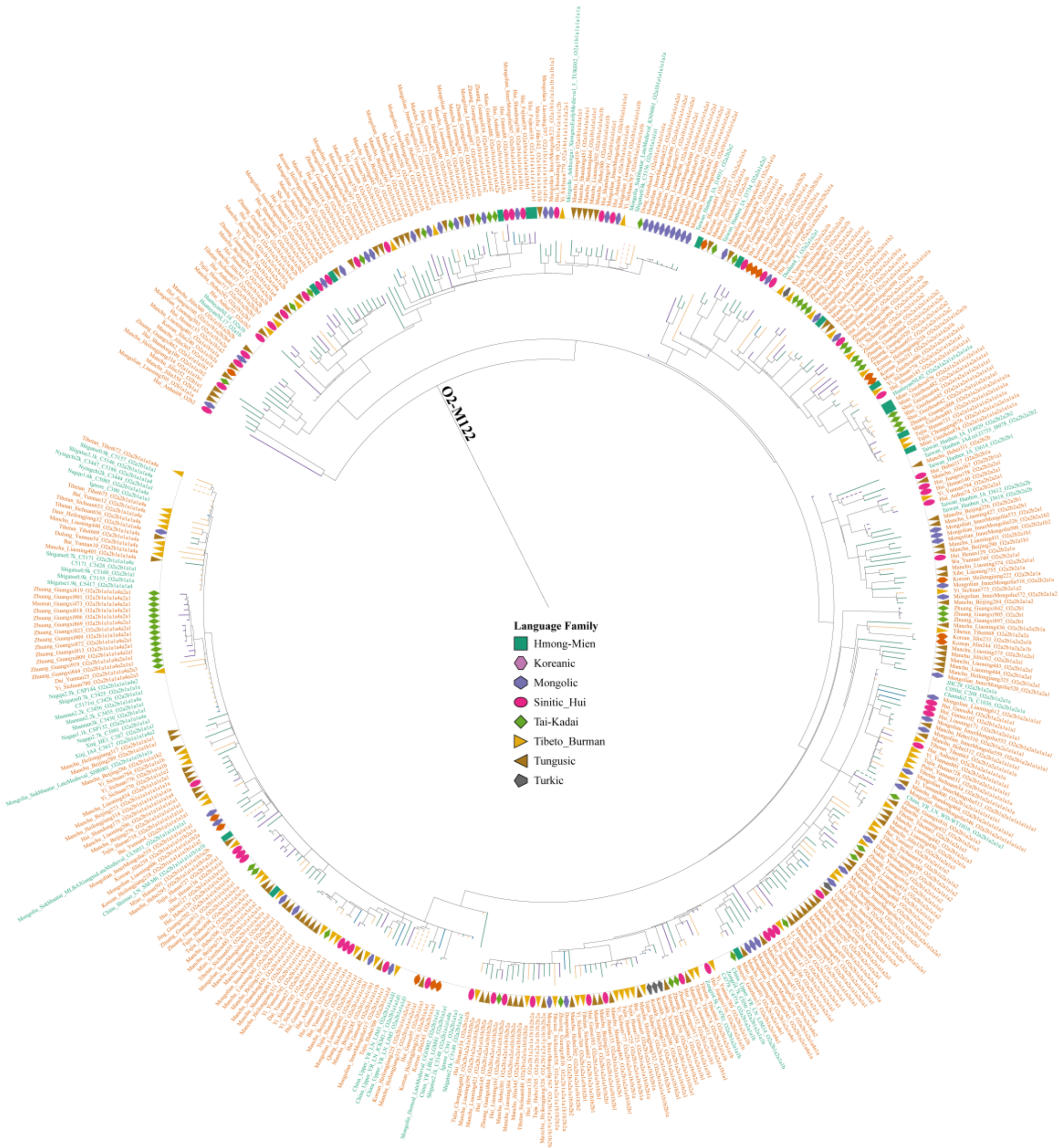

Fig. S12. The detailed O2-related maximum likelihood (ML) phylogenetic tree reconstructed among modern and ancient Eurasian populations. The legend is the same as shown in **Fig. 2**.

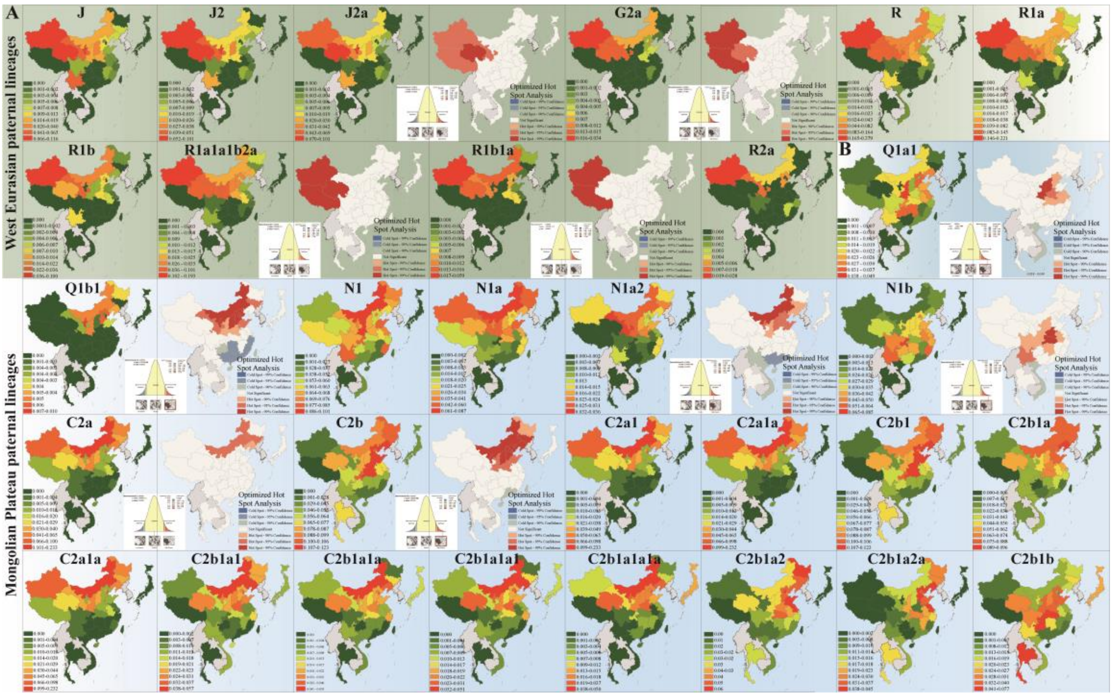

Fig. S13. The HFS of Western Eurasian/Siberian-originated Y-chromosome lineages among modern ethnolinguistically diverse East Asians and Southeast Asians.

(**A**) Haplogroup frequency of western-origin pastoralists and Siberian hunter-gatherer-related lineages among eastern Eurasian populations. (**B**) Haplogroup frequency of sublineages derived from the ancient Siberian related to the fisher and hunter-gatherer people from the Mongolian Plateau, Amur River Basian and Russian Far East. Optimized hot spot analysis (OHSA) suggested the geographical origin of the focused lineages. The hot red color suggested the high-frequency or phylogeographical regions of the studied lineages.

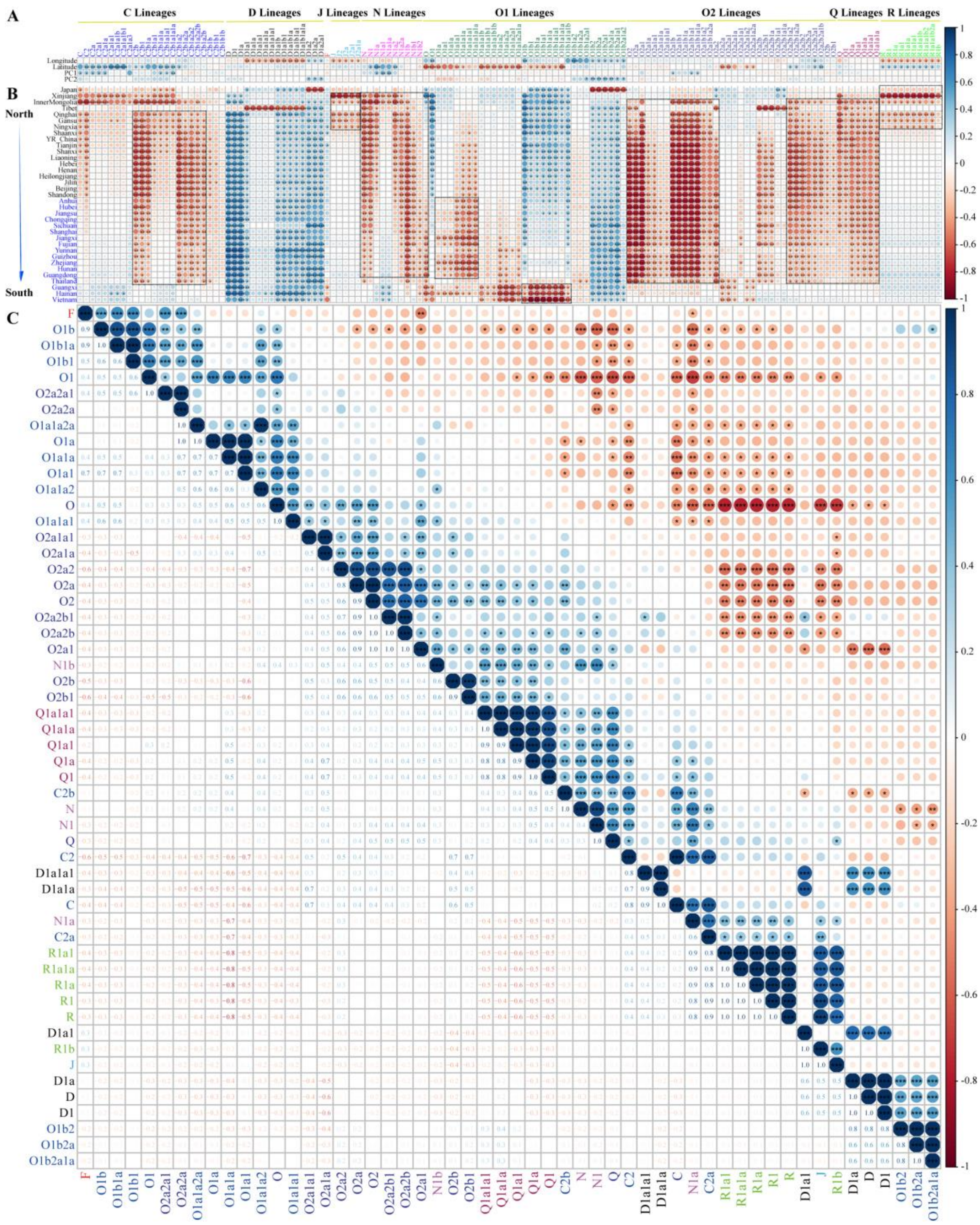

Fig. S14. Correlation between frequency of the Chinese-dominant Y-chromosome lineages and other geographical and genetic features. (**A**) The correlation of frequency of Y-chromosome lineages and geographical features of latitude and longitude, as well as with the top two components of the PCA. (**B**) The correlation between the frequency of Y-chromosome lineages and Fst matrix among eastern Eurasian populations. (**C**) The correlation efficient matrix among the pairwise pairs of haplogroup frequency. Asterisk-labeled tested pairs showed that the Spearman correlation is statistically significant. Three asterisks showed p values less than 0.001; two asterisks showed p values ranging from 0.001 to 0.1, and one asterisk indicated the p values ranged from 0.1 to 0.5. The blue color indicated the positive correlation, and the red color indicated the negative correlation.

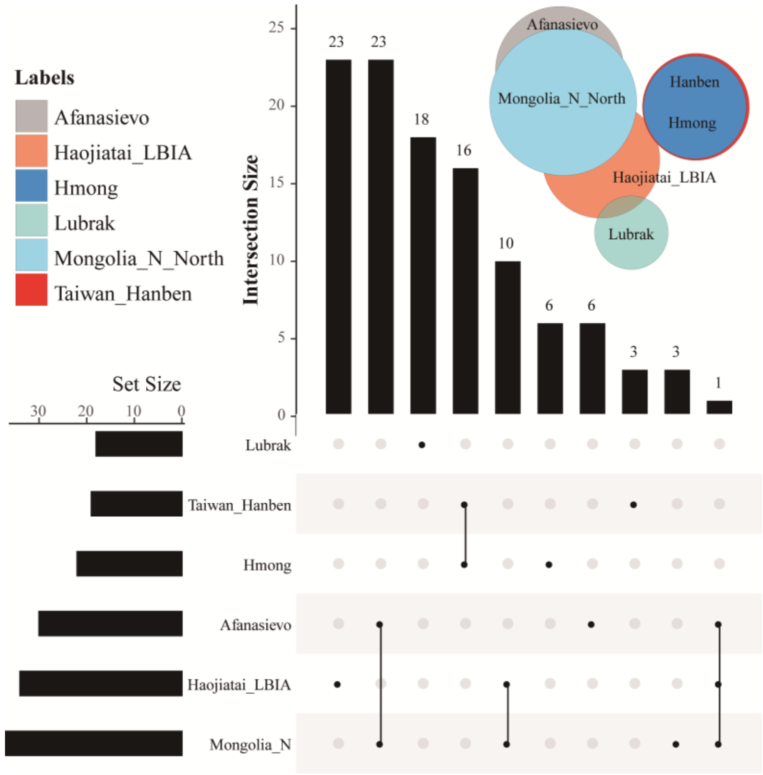

Fig. S15. Venn diagram showed shared and specific lineages among different autosomal-based putative ancestral source-related lineages. The single point refers to a population-specific haplogroup, the two-point line refers to a haplogroup shared by two populations, and the three-point line refers to a haplogroup shared by three populations. The corresponding bar height is the number of haplogroups that are population-specific or shared between populations.

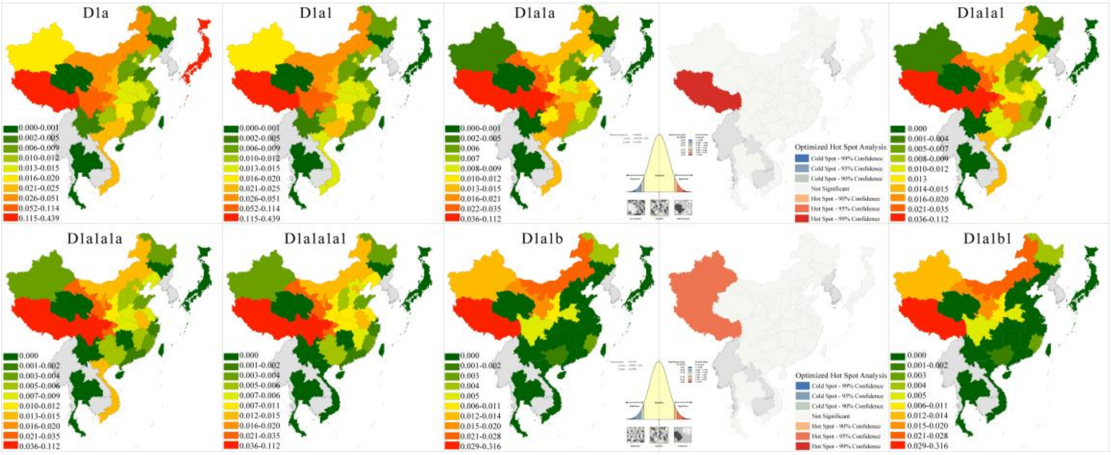

Fig. S16. The HFS of Y-chromosome lineages related to early East Asians among modern ethnolinguistically diverse East Asians and Southeast Asians. Haplogroup frequency of major lineages of D haplogroup. OHSA suggested the geographical origin of the focused lineages. The hot red color suggested the high-frequency or phylogeographical regions of the studied lineages.

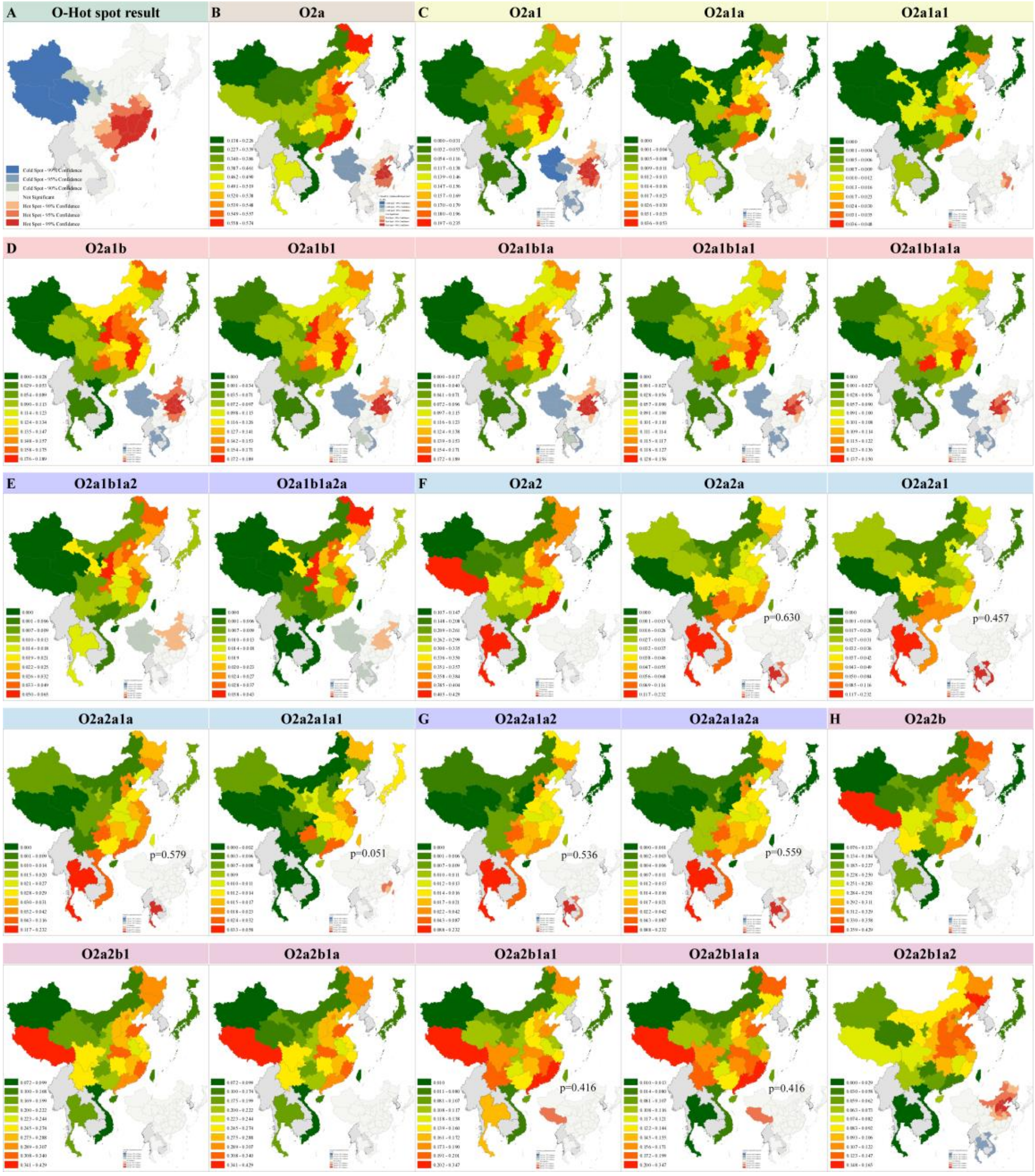

Fig. S17. The HFS of Y-chromosome O2 lineages among modern ethnolinguistically diverse East Asians and Southeast Asians.

(**A**) OHSA result suggested the possible geographical origin of modern East Asians. (**B-G**) Haplogroup frequency of the sublineages of O2. OHSA suggested the geographical origin of the focused lineages. The hot red color suggested the high-frequency or phylogeographical regions of the studied lineages.

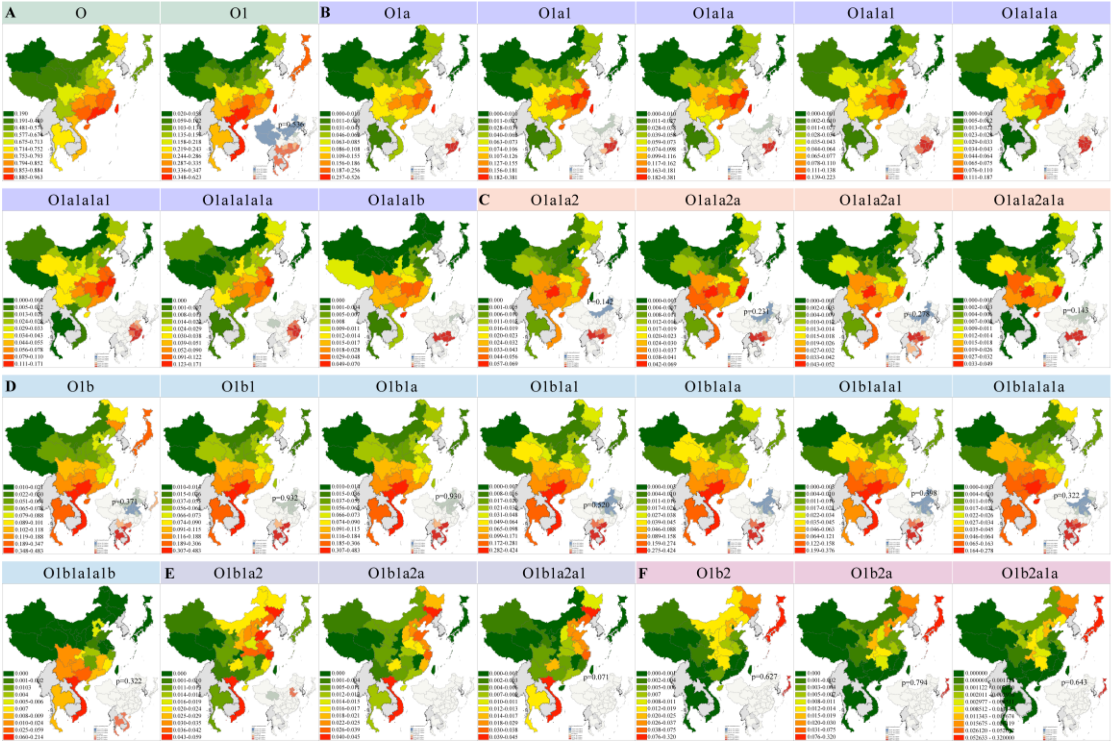

Fig. S18. The HFS of Y-chromosome O and O1 lineages among modern ethnolinguistically diverse East Asians and Southeast Asians. OHSA suggested the geographical origin of the focused lineages. The hot red color suggested the high-frequency or phylogeographical regions of the studied lineages.
